# Genome-wide identification of PIP5K in wheat and its relationship with anther male sterility induced by high temperature

**DOI:** 10.1186/s12870-021-03363-1

**Published:** 2021-12-16

**Authors:** Hongzhan Liu, Zhongke Sun, Lizong Hu, Zonghao Yue

**Affiliations:** College of Life Science and Agronomy, Zhoukou Normal University, Zhoukou, 466001 Henan Province P.R. China

**Keywords:** Wheat, Genome-wide identification, PIP5K, Male sterility, Jasmonic acid isoleucine

## Abstract

**Background:**

Phosphatidylinositol 4 phosphate 5-kinase (PIP5K) plays a key enzyme role in the inositol signal transduction system and has essential functions in plants in terms of growth, development, and stress responses. However, systematic studies on the wheat PIP5K gene family and its relation to male sterility have not been reported yet.

**Results:**

Sixty-four TaPIP5K genes were identified. The TaPIP5K genes contained similar gene structures and conserved motifs on the same branches of the evolutionary tree, and their cis-regulatory elements were related to MeJA-responsiveness. Furthermore, 49 pairs of collinearity genes were identified and mainly subjected to purification selection during evolution. Synteny analyses showed that some PIP5K genes in wheat and the other four species shared a relatively conserved evolutionary process. The expression levels of many conservative TaPIP5K genes in HT-ms anthers were significantly lower than that in Normal anthers. In addition, HT-ms anthers have no dehiscence, and levels of OPDA and JA-ILE are significantly lower at the trinucleus stage.

**Conclusion:**

These results indicate that the PIP5K gene family may be associated with male sterility induced by HT, and the reduction of JA-ILE levels and the abnormal levels of these genes expression may be one reason for the HT-ms anthers having no dehiscence, ultimately leading to the abortion of the anthers.

**Supplementary Information:**

The online version contains supplementary material available at 10.1186/s12870-021-03363-1.

## Background

Plants need to respond to the external environment in which they live. When plants are stimulated by changes in the external environment, extracellular signals need to be transmitted into the cells through the second messenger system, which will cause a series of physiological and biochemical reactions of the cells to successfully adapt to the environment [1]. Phosphatidylinositol and its various derivatives have corresponding signal transmission functions, and the phosphatidylinositol phosphate kinase (PIPK) family converts monophosphorylated phosphoinositol into double phosphorylated phosphatidylinositol derivatives, which play an important role in signal transduction [2, 3]. The phosphatidylinositol 4 phosphate 5-kinase (PIP5K) family belonging to the PIPK family can specifically catalyze the phosphorylation of the hydroxyl group at the D5 position of the 1-phosphatidyl-1D inositol ring to produce phosphatidylinositol (4,5)- Diphosphoric acid (PtdIns(4,5)P2) [4]. This conversion is an important branch in the phosphatidylinositol (PI) cycle. PtdIns(4,5)P2 regulates cell dynamics by regulating the small GTP binding protein ARF and can also interact with related regulatory proteins such as membrane transport and ion membrane transport [5]. Therefore, PIP5K plays a key enzymatic role in the inositol signal transduction system. PIP5K has multiple isoenzymes, which are encoded by a gene family. Compared with animals and fungi, there are relatively more PIP5K genes in the genomes of higher plants. A total of 11 PIP5K genes have been identified in the *Arabidopsis* genome. They have high homology with PIP5Ks (type I PtdInsP kinases) derived from animals. According to their structural differences, that is, whether there is a membrane occupancy, a repeating MORN (membrane occupation and recognition nexus) motif at the N-terminus, and molecular weight, they are further divided into type A (PIP5K10 and PIP5K11) and type B (PIP5K1–9). Based on sequence similarity, the 9 *Arabidopsis* genes of type B are subdivided into 3 subgroups with conservative functions, namely subgroup PIP5K1–3, subgroup PIP5K4–6 and subgroup PIP5K7–9 [6].

PIP5K has multiple isoenzymes and has multiple functions in plants. Studies have shown that the AtPIP5K1 gene of *Arabidopsis* is involved in the ABA signaling pathway, water stress response and pollen development [7, 8]. In addition, the expression of *AtPIP5K1* gene is also induced by drought and high salt stress [7]. The mutant of *AtPIP5K2* gene showed decreased lateral roots and the gravitropism of roots was affected [9]. In addition, the double mutants of *AtPIP5K1* and *ATPIP5K2* showed dwarfism and male sterility [10]. The *AtPIP5K3* gene was specifically expressed in *Arabidopsis* root and could regulate the elongation of root hairs. Subcellular localization showed that the YFP fusion protein was localized on the plasma membrane of the root tip and the protuberant site of the root hairs [11, 12]. *AtPIP5K4* not only regulates stomatal opening [13], but also participates in pollen germination and pollen tube growth together with *AtPIP5K5* gene [14, 15]. Zhao et al. found that the RNA interference of *AtPIP5K6* gene inhibited the growth and development of pollen tubes [16]. It has been reported that the growth of the taproot double mutant plants of *AtPIP5K7* and *AtPIP5K9* is sensitive to polyamine and KCl treatments. Furthermore, it has been reported that Phosphatidylinositol (4,5)-diphosphate (PtdIns(4,5)P2) produced by *AtPIP5K7* and *AtPIP5K9* activate potassium ion efflux in root cells in response to polyamines [17]. Moreover, the recent study of *AtPIP5K7–9* subgroup by Kuroda et al. shows that these genes have little effect on plant growth and development under favorable growth conditions, but they are found to be involved in the adaptation of root growth to osmotic stress. Besides, these genes expressed in other tissues may also participate in the adaptation to stress conditions [17, 18]. *AtPIP5K10* and *AtPIP5K11* genes are specifically expressed in pollen grains and can also play a role in pollen tube growth through actin cytoskeleton recombination [14]. In rice, only one member of the PIP5K family, gene *OsPIP5K1* is known to play a key role in rice heading period [19]. Recently, research on PIP5K gene identification showed that 11, 22 and 12 PIP5K genes were identified in rice, *Glycine max* and *Phaseolus vulgaris*, respectively [20].

Wheat (*Triticum aestivum* L.) is one of the most globally important food crops that supplies vitamins, minerals and protein to humans, accounting for 30% of the land area planted with cereals [21, 22]. The wheat genome is a stable heterohexaploid (AABBDD) gradually formed by the evolution of three subgenomes of A, B, and D. It is large and complex, with a repeat sequence of up to 85% and a size of about 15GB [23]. As an already highly heterozygous allohexaploid, the utilization of wheat heterosis has always been a problem. Although not as successful as in maize and rice, utilization of heterosis is still a favorable way to increase wheat yield on a large scale at present. The creation of male sterile lines is the important premise and foundation of utilizing wheat heterosis [24]. Some reports have shown that multiple components of the PI signaling system are involved in vacuolar changes during pollen development and vesicle transport during pollen tube growth. When these enzymes in the PI signaling system are expressed abnormally, vesicle transport in the pollen tube may affect pollen germination and even cause pollen abortion [25]. The down-regulation of some genes involved in the PI signaling system may be one of the main causes of male sterility, and PIP5K, a key enzyme of the PI signaling system, has not been reported in wheat.

Research on the PIP5K gene family has mainly concentrated on model plants *Arabidopsis* and rice, but there is no report on wheat. With the accurate release of the wheat genome [23], it is possible to analyze the wheat PIP5K gene family. Here, bioinformatic methods were used to identify the members of the wheat PIP5K gene family in the whole genome and a comprehensive analysis of the physical and chemical information, structural functions, and expression patterns of all family members was conducted. The expression patterns of 8 PIP5K genes in wheat anther abortion under high temperature stress conditions was also probed by qRT-PCR experiment. Furthermore, the levels of 12-oxo-phytodienoic acid (OPDA) and active isoleucine jasmonic acid (JA-ILE) content in Normal anthers and sterile anthers were determined. These results will establish a theoretical basis and technical reference for further research on the functional roles of the PIP5K gene family in wheat male sterility.

## Results

### Identification of the PIP5K gene family in wheat

To identify the PIP5K family genes in wheat, the PIP5K HMM profile (pfam: PF01504) was used to search the wheat genome database using blastp, and then the identified proteins sequence was used to reconstruct the wheat-specific PIP5K HMM (*E*-value <1e^− 20^) using hmmbuild. After re-search the wheat genome database using the wheat-specific PIP5K HMM file, 195 candidate PIP5K protein sequences were provisionally identified. 75 candidate PIP5K proteins were eliminated and 120 PIP5K protein sequences were retained after further verification of the existence of PIP5K domains (MORN/PIPKc/PIP5K) in these candidate proteins in PFAM database [26] and SMART database [27]. As many of these protein sequences were translated from the variable transcript of the same gene, we selected the longest transcript sequence as the representative, and named them *TaPIP5K1*-*TaPIP5K64* according to the arrangement of genome A, B and D (Table 1). The identification results of basic physical and chemical properties of PIP5K family members by ExPASY and SignalP showed that the protein sequences of PIP5K family members were quite different (Table 1). The CDS lengths and the protein lengths of the 64 TaPIP5K genes ranged from 1152 to 5520 bp in length and 383 to 1839 amino acids in length, respectively. The predicted protein molecular weights ranged from 44.5 kDa to 205.9 kDa, among which *TaPIP5K20*, *28*, *35* were the largest, and *TaPIP5K*29, 37 were the smallest (Table 1). The theoretical isoelectric points (pIs) ranged from 5.18 (*TaPIP5K15*) to 9.46 (*TaPIP5K18*). Among them, 29 proteins with pIs less than 7 and the pIs of the remaining 35 PIP5K proteins were higher than 7. In addition, all identified PIP5K proteins have no predictable transmembrane domain (Table 1).

**Table 1:** Information about the TaPIP5K genes in wheat

Gene Name	Gene Locus	CDS Length (bp)	AA^a^	MW^b^ (kDa)	pI^c^	TMD^d^
*TaPIP5K1*	TraesCS2A02G130800.1	2349	782	87.9	8.08	0
***TaPIP5K2***	TraesCS2A02G189500.1	4743	1580	176.7	6.40	0
	**TraesCS2A02G189500.2**	4794	1597	178.5	6.37	0
	TraesCS2A02G189500.3	4725	1574	176.1	6.33	0
	TraesCS2A02G189500.4	4782	1593	178.2	6.32	0
	TraesCS2A02G189500.5	4770	1589	177.8	6.36	0
*TaPIP5K3*	TraesCS2A02G479000.1	2412	803	88.9	9.42	0
***TaPIP5K4***	**TraesCS2A02G559200.1**	4464	1487	166.9	5.75	0
	TraesCS2A02G559200.2	4464	1487	166.9	5.75	0
*TaPIP5K5*	TraesCS2B02G152700.1	2343	780	87.5	8.22	0
*TaPIP5K6*	TraesCS2B02G217700.1	4773	1590	175.1	6.31	0
*TaPIP5K7*	TraesCS2B02G503400.1	2406	801	89.1	9.37	0
*TaPIP5K8*	TraesCS2B02G621000.1	4476	1491	167.2	5.66	0
***TaPIP5K9***	**TraesCS2D02G132600.1**	2352	783	87.9	7.84	0
	TraesCS2D02G132600.2	2232	743	83.4	7.24	0
***TaPIP5K10***	TraesCS2D02G198300.1	4815	1604	179.5	6.22	0
	**TraesCS2D02G198300.2**	4899	1632	182.7	6.24	0
	TraesCS2D02G198300.3	4467	1488	167.4	6.25	0
*TaPIP5K11*	TraesCS2D02G478300.1	2406	801	89.0	9.40	0
*TaPIP5K12*	TraesCS2D02G546200.1	2484	827	92.6	9.12	0
*TaPIP5K13*	TraesCS2D02G571300.1	4470	1489	166.5	5.75	0
***TaPIP5K14***	**TraesCS3A02G385500.1**	4848	1615	180.7	5.19	0
	TraesCS3A02G385500.3	4776	1591	178.1	5.25	0
	TraesCS3A02G385500.4	4758	1585	177.5	5.29	0
	TraesCS3A02G385500.5	4680	1559	174.5	5.22	0
	TraesCS3A02G385500.6	4785	1594	178.4	5.27	0
	TraesCS3A02G385500.7	4296	1431	160.5	5.23	0
	TraesCS3A02G385500.8	3402	1133	128.1	6.75	0
	TraesCS3A02G385500.9	3090	1029	116.4	6.12	0
***TaPIP5K15***	TraesCS3B02G417700.1	4791	1596	178.9	5.39	0
	TraesCS3B02G417700.2	4707	1568	175.6	5.31	0
	TraesCS3B02G417700.3	4674	1557	174.3	5.28	0
	TraesCS3B02G417700.4	4752	1583	177.4	5.35	0
	**TraesCS3B02G417700.5**	4869	1622	180.8	5.18	0
	TraesCS3B02G417700.6	4290	1429	160.4	5.29	0
	TraesCS3B02G417700.7	3084	1027	116.3	6.15	0
***TaPIP5K16***	**TraesCS3B02G472100.1**	1731	576	65.2	9.40	0
	TraesCS3B02G472100.2	1596	531	59.5	9.35	0
	TraesCS3B02G472100.3	1605	534	59.9	9.39	0
***TaPIP5K17***	**TraesCS3D02G378500.1**	4848	1615	180.8	5.19	0
	TraesCS3D02G378500.2	4713	1570	175.9	5.25	0
	TraesCS3D02G378500.3	4776	1591	178.2	5.25	0
	TraesCS3D02G378500.4	4680	1559	174.6	5.22	0
	TraesCS3D02G378500.5	4785	1594	178.5	5.27	0
	TraesCS3D02G378500.7	4743	1580	176.9	5.20	0
	TraesCS3D02G378500.8	4296	1431	160.6	5.25	0
	TraesCS3D02G378500.9	3090	1029	116.6	6.16	0
*TaPIP5K18*	TraesCS4A02G087500.1	1590	529	61.0	9.46	0
***TaPIP5K19***	**TraesCS4A02G143600.1**	2238	745	83.9	9.06	0
	TraesCS4A02G143600.2	2235	744	83.8	9.06	0
*TaPIP5K20*	TraesCS4A02G162700.1	5520	1839	205.7	5.83	0
*TaPIP5K21*	TraesCS4A02G165400.1	924	307	35.7	8.13	0
	**TraesCS4A02G165400.2**	1236	411	47.5	9.10	0
*TaPIP5K22*	TraesCS4A02G185600.1	2433	810	90.5	5.84	0
*TaPIP5K23*	TraesCS4A02G271600.1	2187	728	80.7	8.37	0
***TaPIP5K24***	**TraesCS4A02G274200.1**	2412	803	90.2	8.48	0
	TraesCS4A02G274200.2	2043	680	76.1	8.63	0
***TaPIP5K25***	**TraesCS4B02G039400.2**	2298	765	85.8	8.56	0
	TraesCS4B02G039400.3	2262	753	84.7	8.56	0
***TaPIP5K26***	**TraesCS4B02G042400.1**	2193	730	80.8	7.85	0
	TraesCS4B02G042400.2	2190	729	80.7	8.24	0
*TaPIP5K27*	TraesCS4B02G133100.1	2469	822	92.0	5.55	0
*TaPIP5K28*	TraesCS4B02G152100.2	5520	1839	205.7	5.84	0
*TaPIP5K29*	TraesCS4B02G152500.1	1152	383	44.5	8.82	0
***TaPIP5K30***	TraesCS4B02G157800.1	1950	649	72.7	9.18	0
	TraesCS4B02G157800.2	2235	744	83.8	9.00	0
	**TraesCS4B02G157800.3**	2238	745	83.9	9.00	0
*TaPIP5K31*	TraesCS4B02G216900.1	1593	530	61.0	9.40	0
***TaPIP5K32***	TraesCS4D02G036500.3	2220	739	82.3	8.75	0
	**TraesCS4D02G036500.4**	2298	765	85.8	8.61	0
*TaPIP5K33*	TraesCS4D02G039700.2	2187	728	80.6	8.37	0
***TaPIP5K34***	TraesCS4D02G128000.1	2454	817	91.2	5.69	0
	**TraesCS4D02G128000.2**	2556	851	94.9	5.71	0
*TaPIP5K35*	TraesCS4D02G160300.1	5520	1839	205.9	5.91	0
***TaPIP5K36***	TraesCS4D02G161800.1	1950	649	72.8	9.25	0
	**TraesCS4D02G161800.2**	2235	744	83.9	9.09	0
*TaPIP5K37*	TraesCS4D02G161900.1	1152	383	44.5	8.83	0
***TaPIP5K38***	TraesCS4D02G217200.1	1581	526	60.3	9.39	0
	**TraesCS4D02G217200.2**	1599	532	61.1	9.34	0
***TaPIP5K39***	**TraesCS5A02G002500.1**	1587	528	59.1	9.24	0
	TraesCS5A02G002500.2	1578	525	58.7	9.24	0
	TraesCS5A02G002500.3	1578	525	58.7	9.24	0
*TaPIP5K40*	TraesCS5A02G132500.1	2364	787	87.7	6.07	0
*TaPIP5K41*	TraesCS5A02G204600.1	4803	1600	177.2	6.95	0
*TaPIP5K42*	TraesCS5B02G001700.1	1611	536	60.0	9.31	0
*TaPIP5K43*	**TraesCS5B02G132400.1**	2361	786	87.7	6.08	0
	TraesCS5B02G132400.2	2361	786	87.7	6.08	0
*TaPIP5K44*	TraesCS5B02G203700.1	4401	1466	162.5	6.81	0
***TaPIP5K45***	**TraesCS5D02G002700.1**	1620	539	59.9	9.38	0
	TraesCS5D02G002700.2	1218	405	46.3	9.32	0
	TraesCS5D02G002700.3	1611	536	59.5	9.34	0
*TaPIP5K46*	TraesCS5D02G140700.1	2361	786	87.6	6.29	0
*TaPIP5K47*	TraesCS5D02G211500.1	4776	1591	176.0	7.52	0
*TaPIP5K48*	TraesCS6A02G406600.3	2445	814	92.2	8.94	0
***TaPIP5K49***	**TraesCS6B02G451200.2**	2445	814	92.2	8.99	0
	TraesCS6B02G451200.3	2367	788	89.1	8.95	0
*TaPIP5K50*	TraesCS6D02G390100.2	2445	814	92.3	8.88	0
*TaPIP5K51*	TraesCS7A02G205300.1	4806	1601	178.4	5.91	0
*TaPIP5K52*	TraesCS7A02G294300.1	5403	1800	199.3	5.61	0
*TaPIP5K53*	TraesCS7A02G325000.1	4818	1605	179.5	5.61	0
	TraesCS7A02G325000.2	4815	1604	179.4	5.61	0
*TaPIP5K54*	TraesCS7A02G569400.1	2577	858	96.0	9.11	0
***TaPIP5K55***	TraesCS7A02G569800.1	1125	374	43.2	9.22	0
	TraesCS7A02G569800.2	1497	498	56.9	9.26	0
	**TraesCS7A02G569800.3**	1500	499	56.9	9.26	0
***TaPIP5K56***	TraesCS7B02G112700.1	4725	1574	175.1	6.02	0
	**TraesCS7B02G112700.2**	4806	1601	178.0	5.90	0
***TaPIP5K57***	TraesCS7B02G184000.1	5412	1803	199.5	5.72	0
	**TraesCS7B02G184000.2**	5424	1807	200.0	5.69	0
***TaPIP5K58***	**TraesCS7B02G225600.1**	4818	1605	179.5	5.56	0
	TraesCS7B02G225600.2	4815	1604	179.4	5.56	0
*TaPIP5K59*	TraesCS7B02G489300.1	2535	844	94.4	9.13	0
*TaPIP5K60*	TraesCS7D02G208200.1	4806	1601	178.1	5.94	0
*TaPIP5K61*	TraesCS7D02G289700.1	5406	1801	199.5	5.70	0
***TaPIP5K62***	**TraesCS7D02G321500.1**	4809	1602	179.4	5.57	0
	TraesCS7D02G321500.2	4806	1601	179.3	5.57	0
***TaPIP5K63***	**TraesCS7D02G542800.1**	2562	853	96.2	9.04	0
	TraesCS7D02G542800.2	2457	818	92.2	9.07	0
	TraesCS7D02G542800.3	2433	810	91.3	9.04	0
*TaPIP5K64*	TraesCS7D02G543200.2	2568	855	95.4	9.12	0

^a^Length of the amino acid sequence

^b^Molecular weight of the amino acid sequence

^c^Isoelectric point of the TaSTP

^d^Number of transmembrane domains, as predicted by the TMHMM Server 2.0

### Phylogenetic analysis and classification of the PIP5K genes in seven species of dicotyledons and monocotyledons

**Fig. 1:**
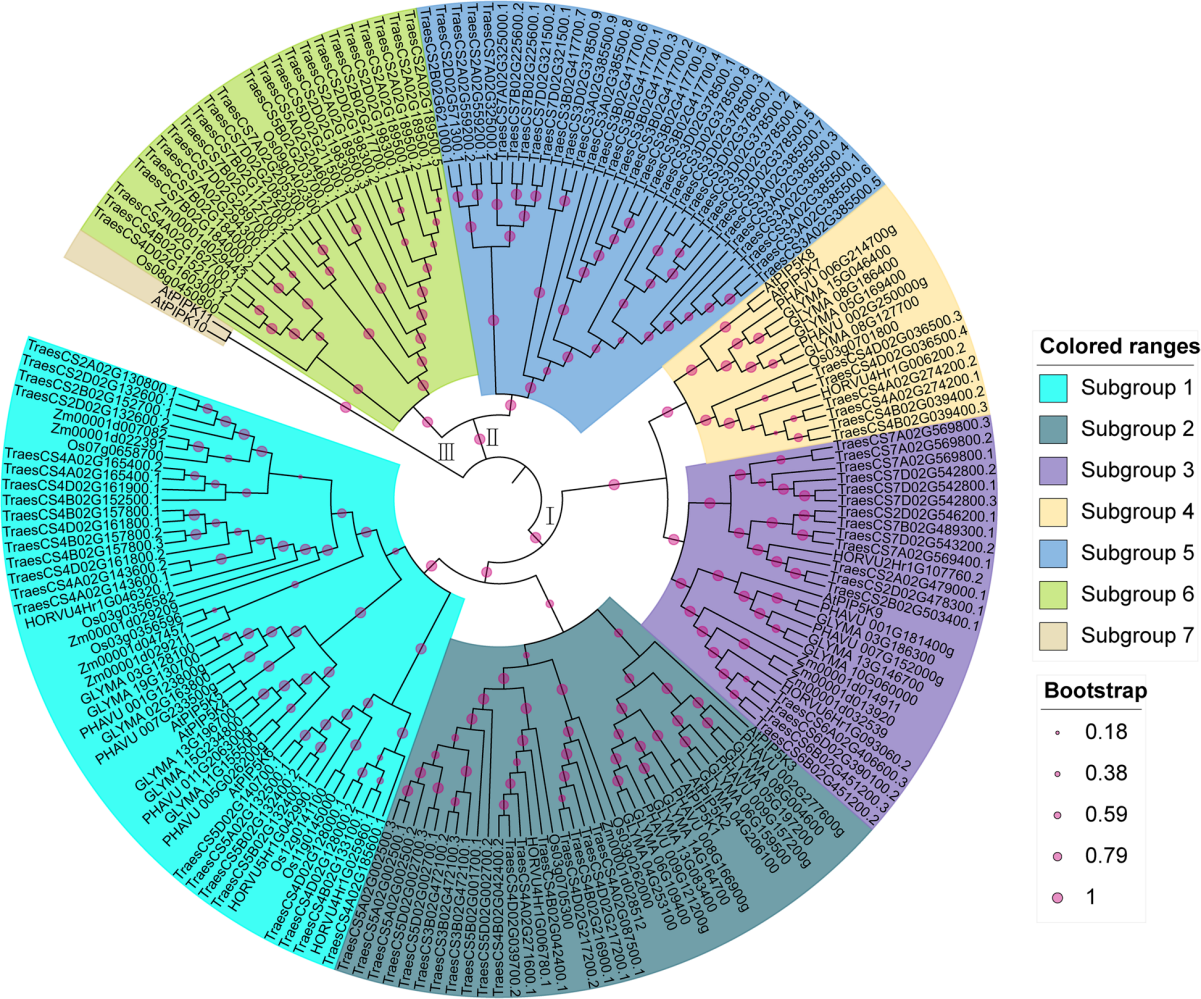
Phylogenetic relationships and subfamily designations of the PIP5K proteins from seven plant species. An evolutionary tree was formed by the phylogenetic relationships of 120 predicted TaPIP5K proteins, 11 *Arabidopsis thaliana* (AtPIP5K1-AtPIP5K9, AtPIPK10-AtPIPK11) proteins, 7 PIP5K proteins in barley, 10 PIP5K proteins in rice, 10 PIP5K proteins in maize, 12 PIP5K proteins in *Phaseolus vulgaris* and 21 PIP5K proteins in *Glycine max* with 1000 bootstrap replicates by MEGA-X. The subgroups are marked with different colors. The new names and accession numbers are shown in Table 1

A phylogenetic tree was constructed according to the 120 PIP5K proteins corresponding to 64 genes in wheat, the 7 PIP5K proteins in barley, the 10 PIP5K proteins in rice, the 10 PIP5K proteins in maize, the 12 PIP5K proteins in *Phaseolus vulgaris*, the 21 PIP5K proteins in *Glycine max*, and the 11 PIP5K proteins (*AtPIP5K1–9*, *AtPIPK10–11*) in *Arabidopsis*. Among them, monocotyledons contain wheat, barley, rice and maize; dicotyledons contain *Phaseolus vulgaris*, *Glycine max* and *Arabidopsis*. The results of the phylogenetic tree show three large branches (I, II, and III) and can be divided into seven subgroups (Fig. 1). Among them, branch I includes subgroup 1–4, which mainly contains *AtPIP5K1–9*, 59 wheat proteins, and all PIP5K proteins in *Glycine max*, barley and *Phaseolus vulgaris*. Branch II includes subgroups 5 and 6, which mainly contain two PIP5K proteins in rice, one PIP5K protein in maize, and 61 PIP5K proteins in wheat. Branch III includes subgroup 7, which are the *Arabidopsis* PIP5K proteins (*AtPIPK10–11*). Branch II is mainly PIP5K protein in wheat, rice and maize. In branch I, it was also shown that all wheat PIP5K proteins are more closely related to rice, maize and barley, and are more distantly related to *Arabidopsis*, soybean and *Phaseolus vulgaris* (Fig. 1).

### Sequence structure features of the TaPIP5Ks

**Fig. 2:**
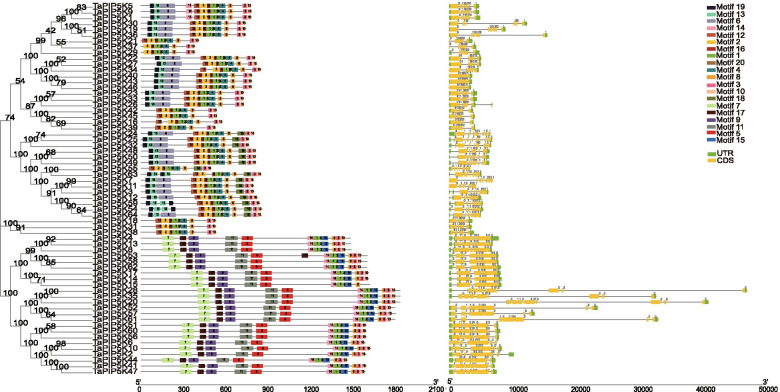
Predicted TaPIP5K protein phylogeny and MEME motif search results and exon-intron structure. The different motifs are represented by different colored boxes numbered at the center of the box and upper right corner of the figure. The non-conserved sequences and introns are shown by black lines in MEME results and exon-intron structure, respectively. The construction method of the phylogenetic tree is the same as that of Fig. 1

In order to understand their functional regions, the online software MEME was used to identify the conserved motifs of wheat PIP5K protein with minimum and maximum motif width set from six to 200. The results of MEME are basically the same as those of the phylogenetic tree (Fig. 2). It was found that all PIP5K proteins contained motifs 1, 3, 8, 10, and 20. Except for the shared motif, most PIP5K proteins in subgroup 1 contain motifs 2, 4, 6, 12, 13, 16 and 19, but *TaPIP5K21*, *TaPIP5K42*, *TaPIP5K45*, *TaPIP5K16*, *TaPIP5K39* and *TaPIP5K55* lack motifs 6, 13 and 19. In addition, *TaPIP5K29* and *TaPIP5K37* also lack motif 12. There is a large branch between the shared motif 8 and motif 3, adding motif 18, such as *TaPIP5K24*, *TaPIP5K25*, and *TaPIP5K32*. Subgroup 2 including *TaPIP5K18*, *TaPIP5K31*, and *TaPIP5K38* also lacks motifs 6, 13, and 19. In addition to the shared motifs, subgroup 3 also contains characteristic motifs 5, 7, 9, 11, 14, 15 and 17. The amino acid distribution of 20 motifs is shown in Fig. S1.

Figure 2 shows that the gene structure of the same subfamily is mutually consistent, and the conserved motifs of the same subfamily are also similar, indicating that genes of the same subfamily have similar functions, such as the branches *TaPIP5K1*, *TaPIP5K5*, and *TaPIP5K9*. All wheat PIP5K genes contain introns, and the longest is *TaPIP5K28*. We analyzed the position of the intron in the gene relative to the 3 nucleotides of the genetic code, that is, the intron phase. If an intron is between two complete codes, the intron is defined as intron phase 0. If the intron is the first and two nucleotides within the codon after that, they are defined as intron phase 1 and intron phase 2, respectively. In subgroups 1 and 2, intron phase 0 and intron phase 1 are almost equally divided, and both contain an intron phase 2. However, subgroup 3 does not contain intron phase 2, but contains eight intron phase 0 and three intron phase 1. In addition, *TaPIP5K21* and *TaPIP5K34* do not contain a UTR area at the 5’end, while *TaPIP5K6*, *TaPIP5K7*, and *TaPIP5K11* do not have a UTR area at the upstream and downstream sides (Fig. 2).

**Fig. 3:**
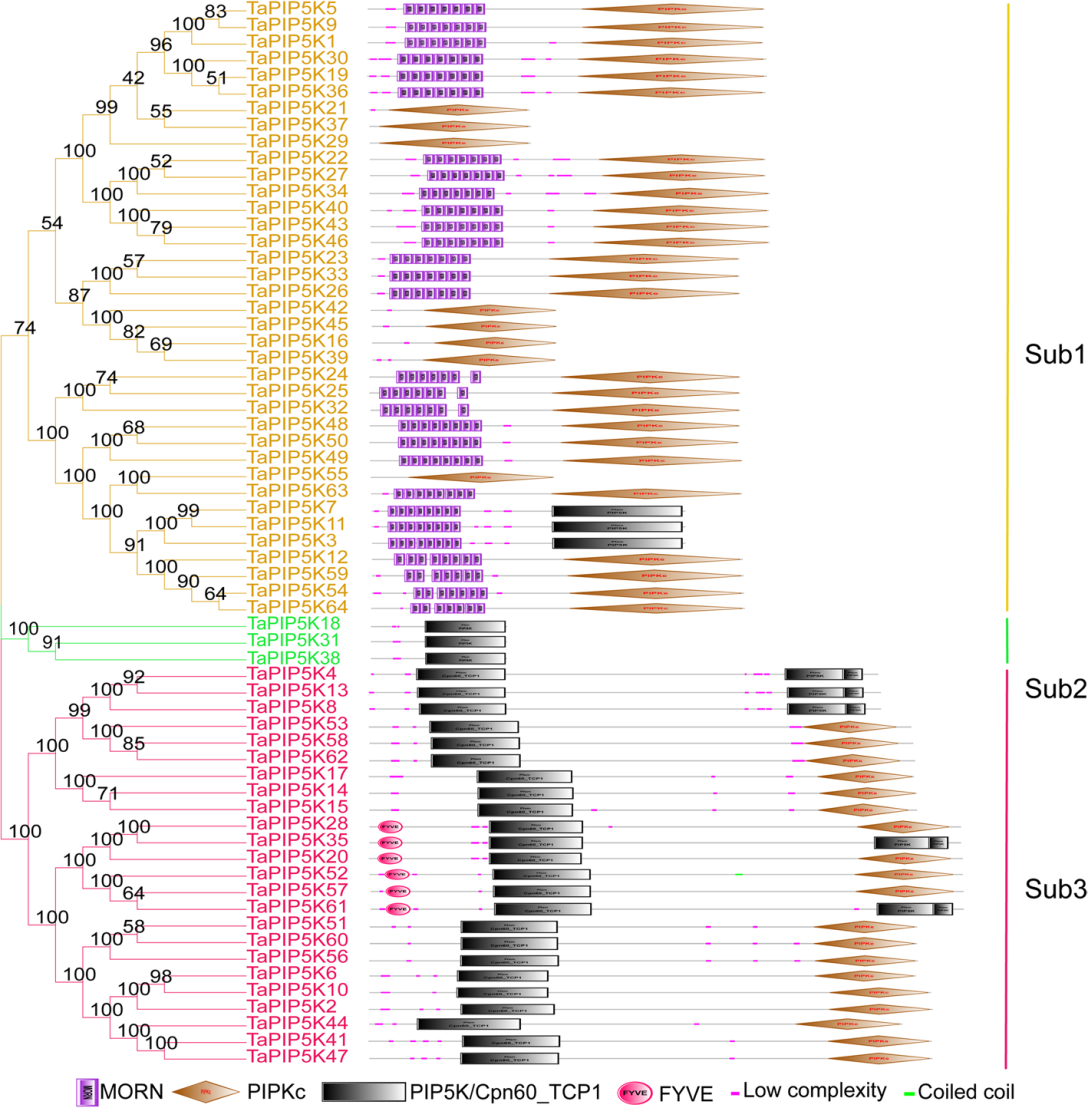
Domain organization of PIP5K genes in wheat. Motif architecture is demonstrated as different shaped and different colored boxes. MORN: membrane occupation and recognition nexus domain, PIPKc: Phosphatidylinositol phosphate kinases domain, FYVE: FYVE domain, Cpn60_TCP1: Chaperonin containing TCP_1 domain, low complexity: low complexity region, coiled coil: coiled coil region

To further clarify the structural domains contained in genes, the SMART website (http://smart.embl-heidelberg.de) was used to analyze the structural domains of TaPIP5K genes in wheat. As shown in Fig. 3, subgroup 1 mainly contains two types of domains, one is the MORN domain, and the other is the PIPKc (Phosphatidylinositol phosphate kinases domain) domain. Among them, 29 TaPIP5Ks (*TaPIP5K1*, *TaPIP5K5, TaPIP5K9, TaPIP5K30,* and so on) contain these two types of domains. Moreover, there are 8 TaPIP5Ks (*TaPIP5K16*, *TaPIP5K21*, *TaPIP5K29*, *TaPIP5K37*, *TaPIP5K39*, *TaPIP5K42*, *TaPIP5K45*, *TaPIP5K55*) that only contain PIPKc domains. Their motif analysis and gene structure analysis both show that they lack some motifs and some gene structures, which is completely consistent with the analysis of the gene domains. In addition, there are 3 genes (*TaPIP5K3*, *TaPIP5K7, TaPIP5K11*) that contain MORN domain and PIP5K domain. Subgroup 2 contains only the PIP5K domain. In addition to the PIPKc domain and the PIP5K domain, an additional Cpn60_TCP1 domain was added in subgroup 3. On this basis, there are 6 genes (*TaPIP5K28*, *TaPIP5K35*, *TaPIP5K20*, *TaPIP5K52*, *TaPIP5K57, TaPIP5K61*) with additional FYVE domains. Interestingly, these domains are accompanied by the emergence of low complexity regions. In the TaPIP5K52, a coiled coil region also appeared between the Cpn60_TCP1 domain and the PIPKc domain.

### Chromosome location and gene duplication of wheat PIP5K gene family

**Fig. 4:**
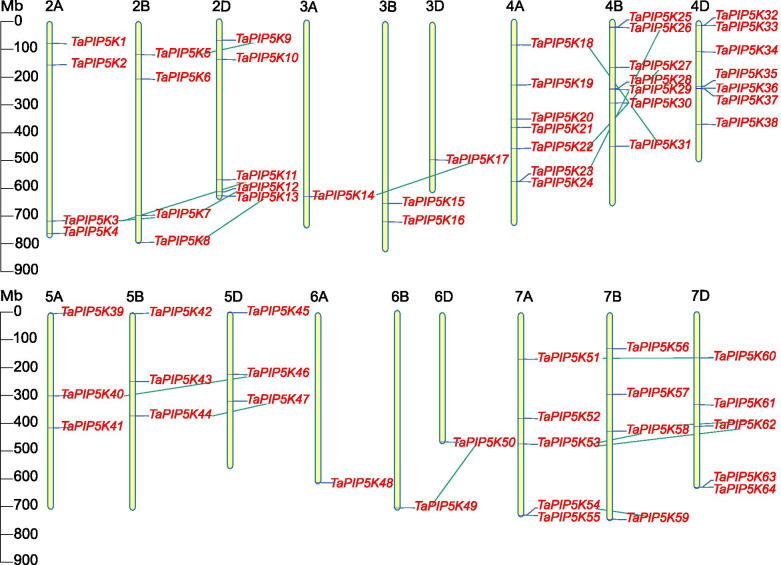
Chromosomal locations of the wheat PIP5K genes. Distribution of the TaPIP5K genes on the wheat chromosomes according to the linkage map. Duplication events are connected by green colored lines. 64 TaPIP5K genes were mapped to 18 chromosomes (2A-7A, 2B-7B and 2D-7D). The scale is in megabase pair (Mbp). Six gene clusters containing two genes were distributed on the 4A, 4B, 4D, 7A and 7D chromosomes, respectively

Wheat PIP5K genes are unevenly distributed on 18 of 21 wheat chromosomes, and no PIP5K genes are found on chromosomes 1A, 1B, and 1D. There are 7 (in 4A, 4B, 4D), 5 (in 2D, 7A, 7D), 4 (in 2A, 2B, 7B) and 3 (in 5A, 5B, 5D) PIP5K genes in different chromosomes, respectively”. 3B chromosome contains 2 genes, and the remaining chromosomes contain one gene. Fifty percent (34/64) of wheat PIP5K members showed repetitive events. There were no tandem repeats in these repetitive events, but the presence of highly similar genes on different chromosomes indicated that fragment repetitive events occurred.. As shown in Fig. 4, the link regions of segment duplications on chromosomes 4A, 4B and 4D all occur between chromosomes 4A and 4B. There are all 5 segment duplication events that occur unequally between chromosomes A, B, and D on chromosomes 2A, 2B, 2D and chromosomes 7A, 7B, and 7D, respectively. The least are 3A, 3B, 3D chromosomes and 6A, 6B, 6D chromosomes, each of which has only one segment duplication event.

Furthermore, we found that all homologous genes are in the same evolutionary branch combined with phylogenetic tree cluster analysis, e.g., evolutionary branches *TaPIP5K3*, *TaPIP5K7*, *TaPIP5K11* and evolutionary branches *TaPIP5K53*, *TaPIP5K58*, *TaPIP5K62* in 3 partial homologous chromosomes (A, B, D) all have homologous sites, and they are located on chromosomes 2A, 2B, 2D and 7A, 7B, 7D, respectively (Figs. 3 and 4). The other homologous genes have homologous sites on two partial homologous chromosomes (A or B, A or D, B or D), indicating that the wheat PIP5K gene has a large number of homologous sites, showing a high homology retention rate. Moreover, we found 6 gene clusters composed of 2 genes, namely *TaPIP5K23* and *TaPIP5K24* on chromosome 4A; TaPIP5K25 and TaPIP5K26 on chromosome 4B; TaPIP5K32 and TaPIP5K33, TaPIP5K36 and TaPIP5K37 on chromosome 4D; TaPIP5K54 and TaPIP5K55 on chromosome 7A. TaPIP5K63 and TaPIP5K64 on chromosome 7D (Fig. 4).

**Fig. 5:**
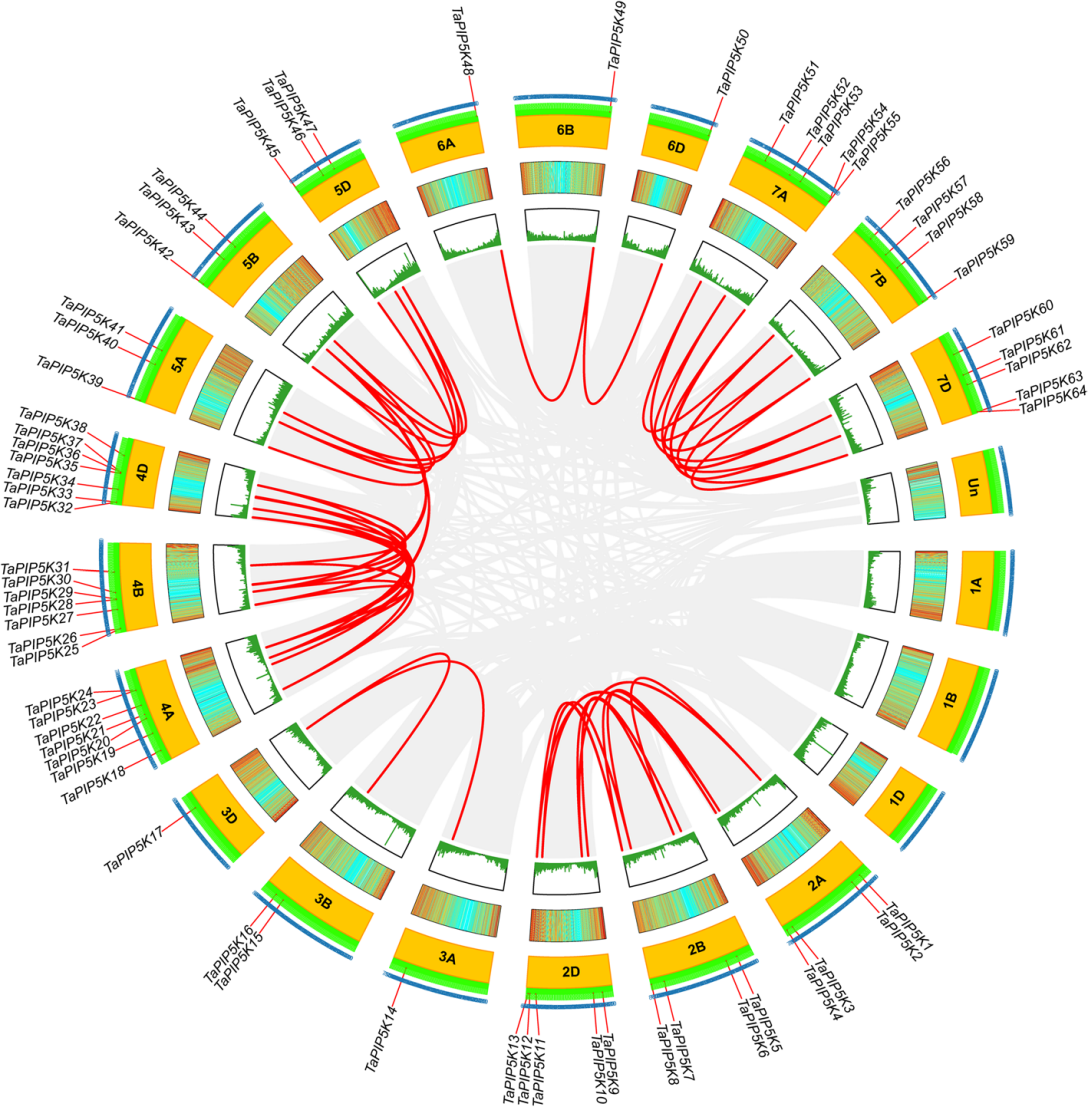
Localization and synteny of the TaPIP5K genes in the wheat genome. The TaPIP5K genes in wheat were mapped to different chromosomes. The chromosome number is indicated on the inside with a yellow color. Gene pairs of the TaPIP5K with a syntenic relationship are joined by a red line. The TaPIP5K gene names are located on the outermost side of the chromosome. Gray lines in the background indicate the other gene pairs within wheat

The Bio-linux system was used to further analyze the collinearity of these PIP5K genes between wheat chromosomes through the two-way blast comparison and the MCScanX tool (the ones connected by the red line are the collinearity genes), and a total of 49 pairs of collinearity genes were identified. Gene pairs with a syntenic relationship are joined by a red line. The results are shown in the Circos diagram (Fig. 5), and the detailed data is shown in Table S1. These collinearity genes are more of the paralogous genes that appear in the same chromosome group (2A, 2B, 2D; 3A, 3B, 3D; 6A, 6B, 6D; 7A, 7B, 7D). Between 4A, 4B, 4D and 5A, 5B, 5D, in addition to the paralogous genes in the same chromosome group, there also appeared cross-chromosomal paralogous gene replication events.

Gene duplication in plants mainly includes fragment duplication and tandem duplication, and duplication events are crucial to the amplification of gene family members during plant evolution. In detail, the TaPIP5K43 gene on chromosome 5B has a collinearity relationship with TaPIP5K22 gene on chromosome 4A and TaPIP5K27 gene on chromosome 4B, respectively (Fig. 5). This also indicates that chromosomes 4A, 4B and 5B may have experienced chromosome fragment duplication. Chromosomes 4A, 4B and 5B are also chromosomes with more paralogous genes, so these chromosomes may be the key chromosomes of the TaPIP5K gene family. This also indicates that fragment replication events may be the main reason for the amplification of members of the wheat PIP5K gene family (Additional file 1).

**Fig. 6:**
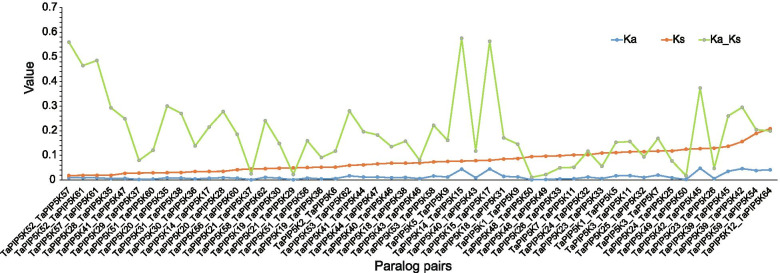
The Ka/Ks value of paralog pairs of TaPIP5K in wheat. The blue line refers to Ka values, the brownish yellow line refers to Ks values, and Ka/Ks values are shown by a light green line. The vertical line and horizontal line indicate the values of Ka, Ks, Ka/Ks and paralog pairs, respectively

In the biological evolution process, non-synonymous mutations refer to gene mutations that can cause changes in the amino acid sequence of a polypeptide product or functional RNA base sequences; gene mutations that do not cause changes in the amino acid sequence are synonymous mutations. The ratio of non-synonymous mutation frequency (Ka) to synonymous mutation frequency (Ks) (Ka/Ks) is less than 1, equal to 1 or greater than 1, indicating that the gene is subject to purification selection, gene neutral evolution, or gene positive selection, respectively. In order to further study the selection of these replicated gene pairs, after calculating the Ka/Ks ratio, it was found that the Ka/Ks values of all wheat PIP5K genes were less than 1, and the maximum value was 0.575. The corresponding gene pair was *TaPIP5K14* and *TaPIP5K15* (Fig. 6, Additional file 2).

### Synteny analysis of TaPIP5K genes between wheat and four representative plant species

**Fig. 7:**
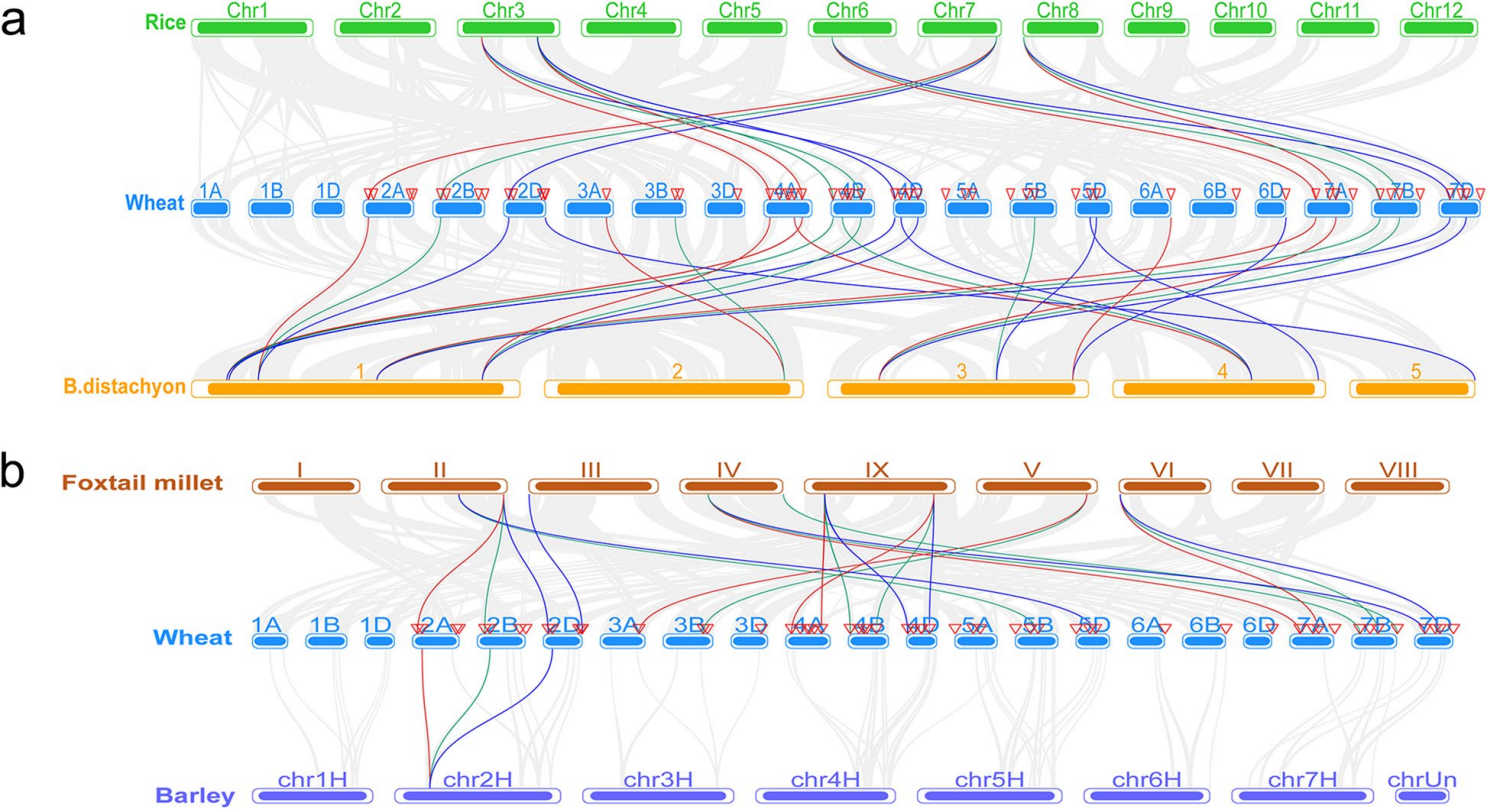
Synteny analysis of TaPIP5K genes between wheat and four representative plant species. Gray lines in the background indicate the collinear blocks within wheat and other plant genomes, while the other red, green and blue colored lines highlight the collinear blocks located in A, B and D chromosome in wheat, respectively. The species names ‘Wheat’, ‘Rice’, ‘B.distachyon’, ‘Foxtail millet’, and “Barley” indicate *Triticum aestivum*, *Oryza sativa*, *Brachypodium distachyum*, *Setaria italica*, and *Hordeum vulgare*, respectively. The chromosomes of different species are colored differently. **a** Synteny analysis of TaPIP5K genes between wheat and rice and *B. distachyon*; **b** Synteny analysis of TaPIP5K genes between wheat and foxtail millet and barley

In order to further understand the evolutionary mechanism of the PIP5K gene family, we constructed a synteny map of the PIP5K family of wheat and rice, *Brachypodium distachyum*, foxtail millet, and barley (Fig. 7; Additional file 3). These four plants are monocotyledonous and closely related to wheat. Finally, it was identified that wheat and rice have 15 pairs of orthologous genes, and 26 pairs of orthologous genes with *Brachypodium distachyum*. These orthologous genes are in every three on rice and are located on the same chromosome position, while on *Brachypodium distachyum*, there are one, two or three orthologous genes distributed unevenly on 12 sites on five chromosomes (Fig. 7a). The collinearity analysis of wheat, foxtail millet and barley found that there are 21 pairs of orthologous genes in wheat and millet, which are unequally distributed in 9 loci on 9 chromosomes of foxtail millet with 1 to 3. There are only 3 pairs of orthologous genes, distributed in the HORVU2Hr1G036380.5 site of chr2H, corresponding to the 2A, 2B and 2D chromosomes of wheat, respectively (Fig. 7b). The orthologous genes of wheat, rice, *Brachypodium distachyum*, foxtail millet and barley are more widely distributed on 4A, 4B, 4D and 7A, 7B, 7D, especially in rice and *Brachypodium distachyum*. These chromosomal sites in wheat have orthologous genes in both plants, and they all have similar collinearity blocks.

### Analysis of cis-elements of wheat PIP5K gene family

**Fig. 8:**
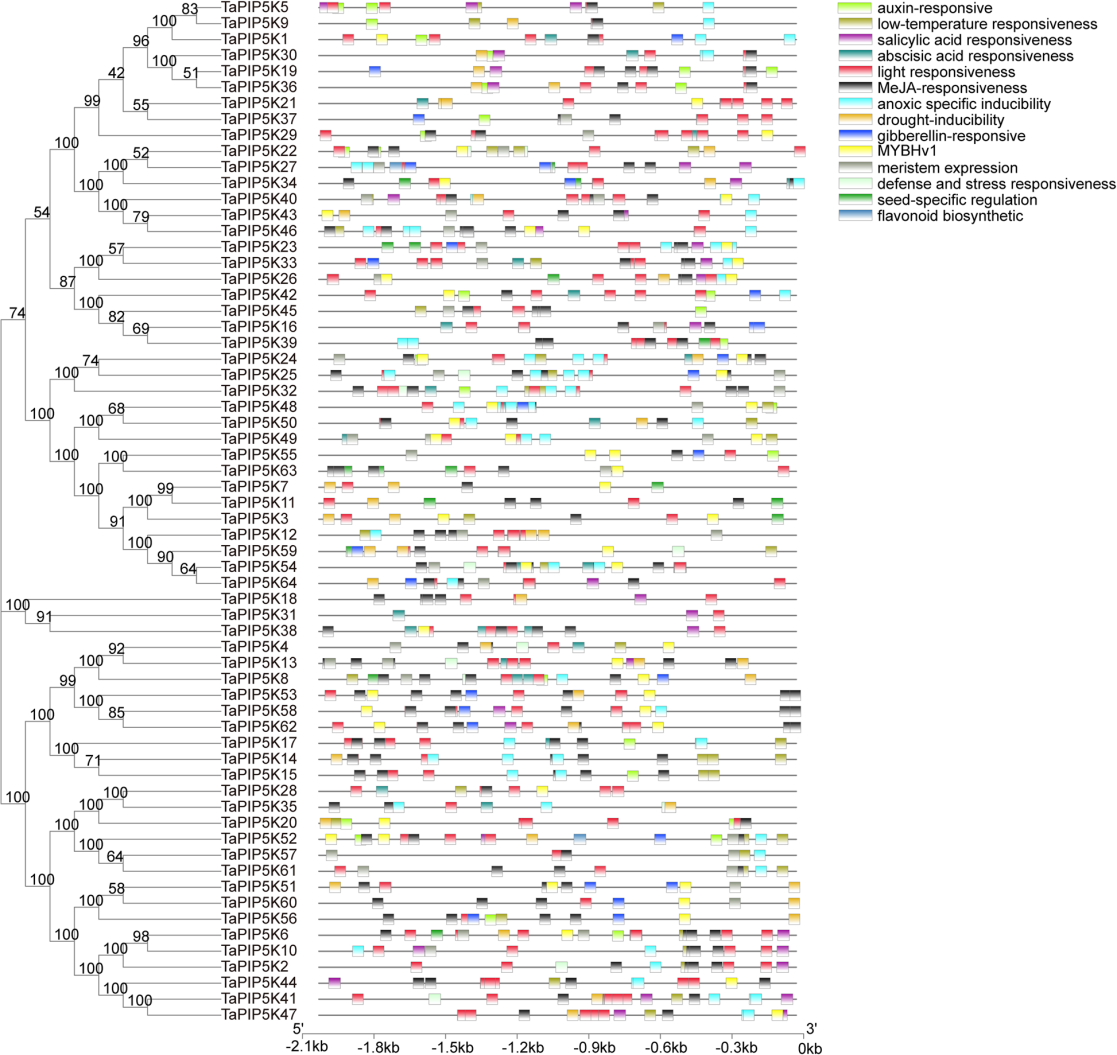
Cis-elements. Putative cis-elements were detected in 2-kb promoters of wheat pip5k genes. The horizontal axis indicates region nucleotide length of gene promoter; the color bar code represents the different cis-elements in the promoter region. The phylogenetic tree is the same as that of Fig. 2

In order to explore the factors affecting the expression of PIP5K gene and the regulatory pathways that PIP5K gene may participate in, PlantCARE and TBtools were used to analyze the regulatory sequence of the 2 kb region upstream of the translation initiation site (ATG). 106 cis-elements have been identified from wheat PIP5Ks. In addition to the traditional promoter elements (TATA-box, CAAT-box), 14 of them are either detected or have special significance in their functions. These 14 promoter elements can be basically divided into four groups: 4 are hormone-responsive, including MeJA-responsiveness (TGACG-motif, CGTCA-motif), auxin-responsive (TGA-element), abscisic acid responsiveness (ABRE), gibberellin-responsive element (P-box, GARE-motif, TATC-box); 2 types are stress response elements, including drought-inducibility (MBS), defense and stress responsiveness (TC-rich repeats). The third group is expression-related cis-acting elements, such as seed-specific regulation (RY-element), merge expression (CAT-box), etc. The other group is other cis-acting elements, such as light responsiveness (ACE), low-temperature responsiveness (LTR), salicylic acid responsiveness (TCA-element), anaerobic induction (ARE), flavonoid biosynthetic genes regulation (MBSI), and MYBHv1 (CCAAT-box) (Fig. 8). Except for *TaPIP5K21* and *TaPIP5K31*, the MeJA-responsiveness promoter is present in all the other identified genes. The least of which contains only one MeJA-responsiveness promoter (*TaPIP5K1, TaPIP5K5, TaPIP5K9, TaPIP5K20, TaPIP5K31, TaPIP5K57*). The *TaPIP5K53* gene contains 6 MeJA-responsiveness promoters, and there are several MeJA-responsiveness promoter elements on some genes appearing together, such as *TaPIP5K12, TaPIP5K18, TaPIP5K32, TaPIP5K53, TaPIP5K58, TaPIP5K60*, and others. Light responsiveness promoter elements are present in all identified TaPIP5K genes, the number ranging from 1 to 5. Some genes also have light responsiveness promoter elements appearing in succession, such as *TaPIP5K21, TaPIP5K41, TaPIP5K47*, and others. The details of the other promoters are shown in Additional file 4. These results suggest that the PIP5K gene may be involved in the response process of plants to hormones and adversity stress. Furthermore, some homologous genes have similar cis-regulatory elements, such as *TaPIP5K41*,*47* and *TaPIP5K14*,*15*,*17*. There are differences between genes of different branches, such as the number of elements, which implies that some of the different TaPIP5K genes are regulated by common factors and show similar expression patterns in wheat. Some TaPIP5K genes have differences in regulation and expression, so that they can play a role in a variety of physiological processes.

### Tissue expression analysis of wheat PIP5K genes

**Fig. 9:**
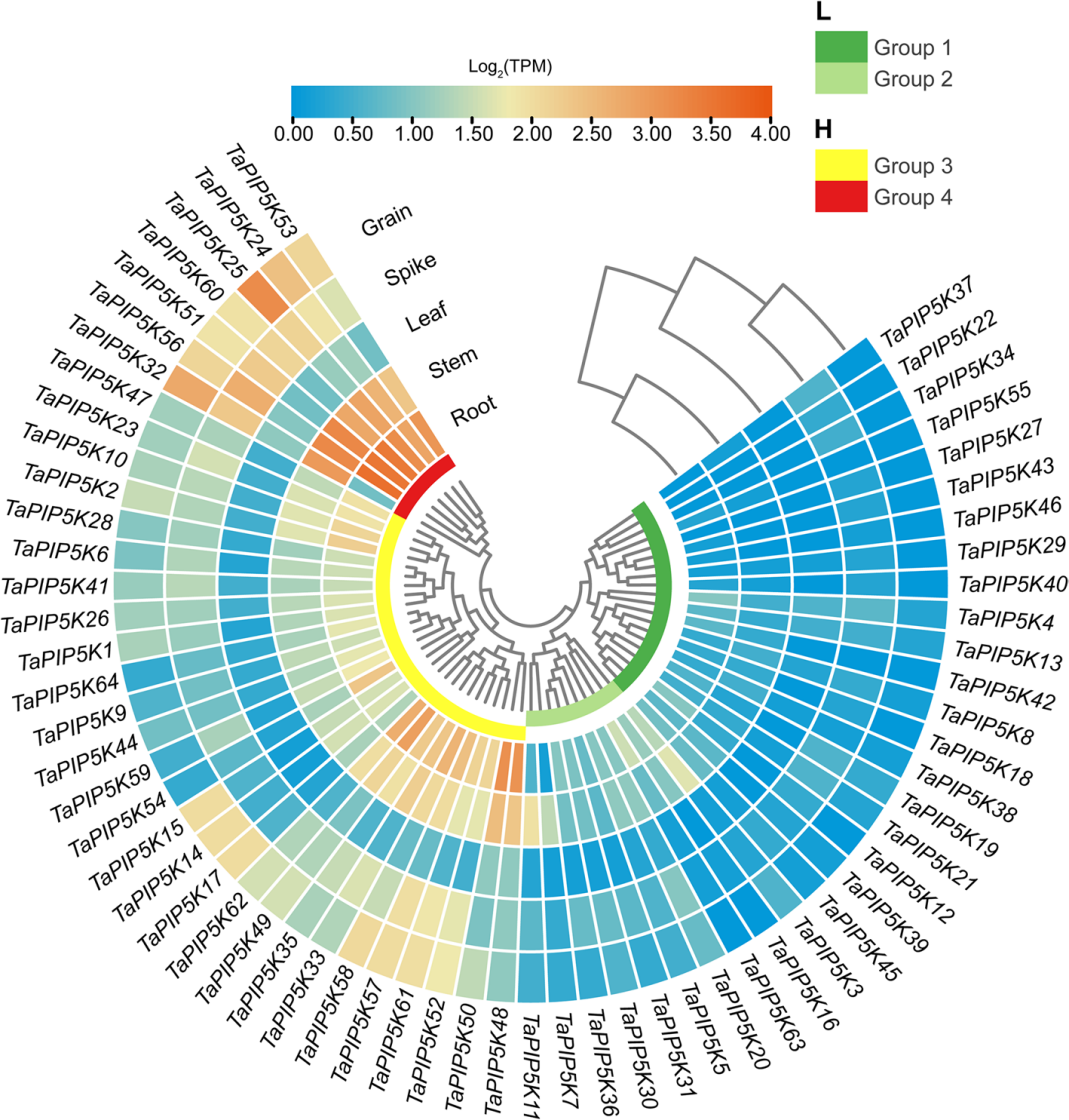
Expression patterns of PIP5K family genes in different tissues in wheat. Expression profiles of TaPIP5Ks in different tissues obtained from RNA-seq data reported in WheatOmics. The transcriptome expression data of different tissues in the wheat variety China Spring were downloaded, and the transcripts per kilobase million (TPM) values of TaPIP5Ks gene were screened. Results are shown as a heat map in blue/yellow/brownish red (low to high) that reflect the value of log^2^(TPM) of expression

Using the RNA-seq data of wheat variety China Spring, the expression of TaPIP5K in different tissues was analyzed. According to the heat map results, the expression level of 64 TaPIP5K genes are generally low in wheat leaves, while the expression levels of different genes in roots, stems, spikelets, and grains vary greatly (Fig. 9, Additional file 5). We divided the 64 genes into two major categories according to cluster analysis, namely low expression region (L) and high expression region (H). Low expression region included group1 and group2. The genes involved in group1 are at low expression levels in the five tissues. The expression of group2 has increased relative to the expression of group1, and the expression of individual genes has increased significantly. For instance, the expression of *TaPIP5K3* and *TaPIP5K11* in stems is significantly higher than that in other tissues. The high expression regions include group3 and group4. The TaPIP5K genes of group3 showed a tendency of high expression in roots and stems, especially *TaPIP5K48*, *TaPIP5K49*, *TaPIP5K50*, *TaPIP5K58*, and *TaPIP5K62*. *TaPIP5K*33, *TaPIP5K52*, *TaPIP5K57* and *TaPIP5K61* were highly expressed in spikelets, while *TaPIP5K14*, *TaPIP5K15*, *TaPIP5K17*, *TaPIP5K57*, *TaPIP5K58*, *TaPIP5K61* and *TaPIP5K62* were highly expressed in grains. The TaPIP5K genes (except *TaPIP5K*32) of group 4 were highly expressed in roots, stems, spikelets, and grains. The above results indicate that the expression patterns of some TaPIP5K genes in different tissues are similar, but some genes are also very different, suggesting that TaPIP5K genes play an important role in the growth and development of wheat. It also implies that a certain degree of biological function differentiation may have occurred between different TaPIP5K members.

### The relationship between wheat PIP5K gene family and high temperature induced anther sterility and qRT-PCR analysis

**Fig. 10:**
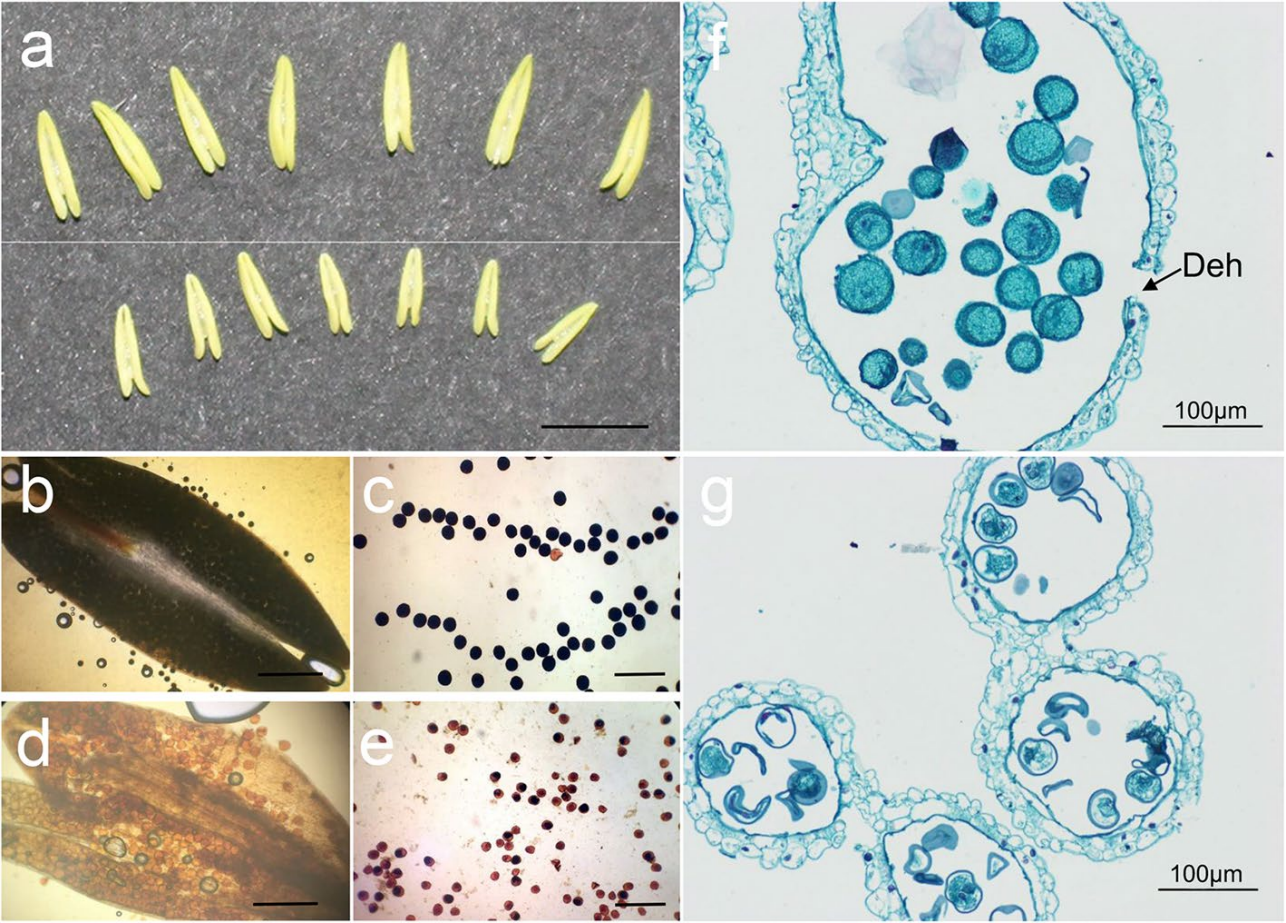
Comparison between appearance and transections of the trinuclear stage anthers and pollen grains in Normal and HT-ms plants. **a** The morphology of Normal (above the white line) and HT-ms (below the white line) anthers at the trinuclear stage. Anther (**b**) and pollen grains (**c**) from a Normal plant stained with KI-I_2_ solution. Anther (**d**) and pollen grains (**e**) from a HT-ms plant stained with KI-I_2_ solution. **f** Cross sections of a Normal locule at the trinuclear stage of development. **g** Cross sections of a HT-ms locule at trinuclear stage of development. Cross sections were stained for 20 min with 1% SafraninO, followed by staining for 40s–60s with 1% Fast Green. Deh, dehiscence. Bars = 2 cm (**a**), 200 μm in (**b** and **d**), 100 μm in (**c, e, f, g**)

In the previous study, it was confirmed that wheat undergoes appropriate high temperature stress during the period of female stamens primordia formation, which can lead to complete male sterility in anthers [28]. Compared with Normal anthers, high temperature induced sterile anthers (HT-ms anthers) have very significant characteristics. One is that they are smaller and thinner compared with Normal anthers (Fig. 10a). The other is that Normal anthers have sufficient starch accumulation. KI-I_2_ staining of whole anthers and pollen grains were shown completely black in Normal anthers (Fig. 10b, c), but shown light yellow in whole anthers and pollen grains in HT-ms anthers, namely no starch accumulation or less starch accumulation (Fig. 10d, e). In addition, we compared the anther dehiscence phenomenon in the HT-ms anthers with that in the Normal anthers. The HT-ms anthers had no dehiscence, which is consistent with the obvious dehiscence of the Normal anthers at the trinuclear stage shown by the safranine fast green stained sections (Fig. 10f), while the HT-ms anthers at the trinuclear stage did not display this phenomenon (Fig. 10g).

**Fig. 11:**
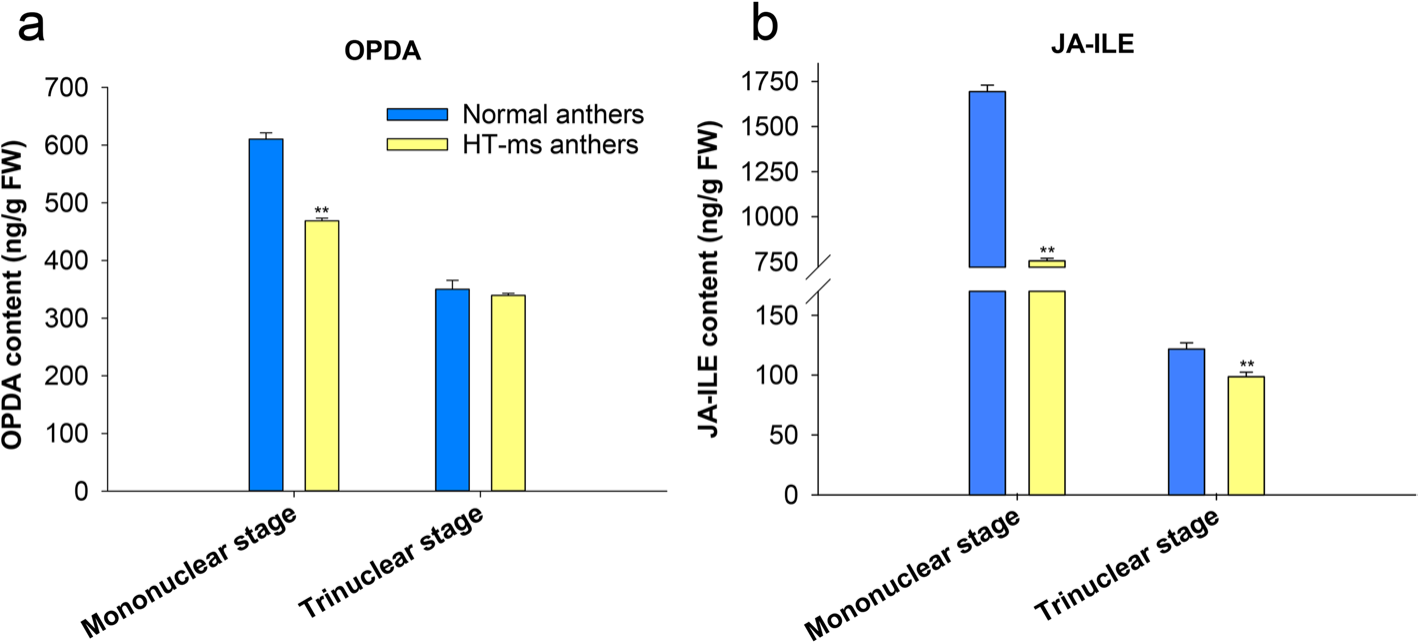
The OPDA and JA-ILE content in Normal and HT-ms anthers was measured at the mononuclear and trinuclear stage of development. **a** The content of OPDA. **b** The content of JA-ILE. Mix sample was used for three independent replicates (*n* = 3). *, ** = significantly different from the Normal anthers control at *p* < 0.05 and *p* < 0.01, respectively

Anther dehiscence is closely related to jasmonic acid-related hormones, and our cis-element results also show that most of the upstream promoter regions of the wheat PIP5K gene family have MEJA-response and light responsiveness-related elements. We further quantified the levels of 12-oxyphytodienoic acid (OPDA, a key substance in the synthesis of jasminin), and JA-Ile in HT-ms anthers and Normal anthers at mononuclear and trinuclear stages. The determination of OPDA showed that OPDA content of Normal anthers was higher than that of HT-ms anthers at mononuclear and trinuclear stages. In detail, the OPDA content in the Normal mononuclear anthers was 0.30 times higher than in the HT-ms mononuclear anthers showing significant difference. The OPDA contents were similar in the Normal and the HT-ms anthers at trinuclear stage showing no significant difference (Fig. 11a). The active form of jasmonic acid in the organism is jasmonic acid isoleucine (JA-ILE). Compared with Normal anthers, it was showed that the JA-ILE content was 1.24 times higher and 0.23 times higher than HT-ms anthers at mononuclear stage and trinuclear stage, respectively (Fig. 11b, see Additional file 6 for details).

### Expression of wheat TaPIP5K genes in Normal and HT-ms anthers at mononuclear stage and trinuclear stage

**Fig. 12:**
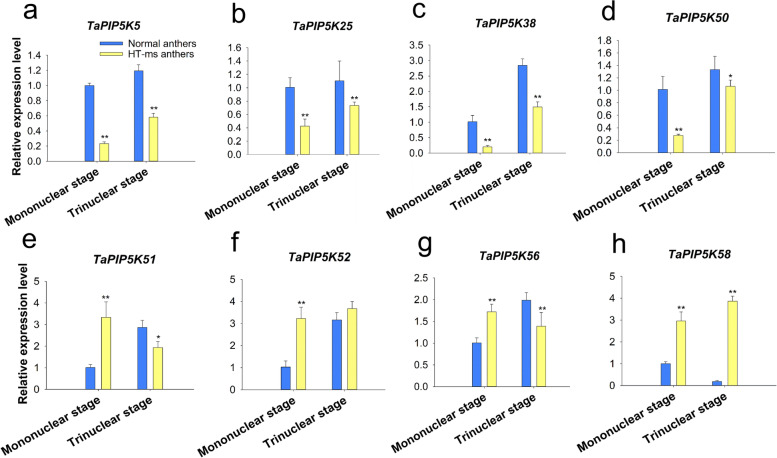
Comparison of the relative expression levels of eight TaPIP5Ks involved in Normal and HT-ms anthers at the mononuclear and trinuclear stage of development. The x-axes indicate the relative gene expression levels; the y-axes indicate the different stage in the Normal and HT-ms anthers. a to h show the relative expression levels of *TaPIP5K5*, *TaPIP5K25*, *TaPIP5K38*, *TaPIP5K50*, *TaPIP5K51*, *TaPIP5K52*, *TaPIP5K56*, and *TaPIP5K58* genes in Normal and HT-ms anthers at the mononuclear and trinuclear stage, respectively. Capped lines indicate standard error. * *P* < 0.05; ** *P* < 0.01

We investigated the expression levels of the TaPIP5K genes in Normal and HT-ms anthers at mononuclear and trinuclear stages to further explore TaPIP5K gene functions in wheat. First, according to the grouping in Fig. 9, we randomly selected eight genes with a particular focus on the highly expressed genes in spikelets, *TaPIP5K5*, *TaPIP5K25*, *TaPIP5K38*, *TaPIP5K50*, *TaPIP5K51*, *TaPIP5K52*, *TaPIP5K56*, and *TaPIP5K58,* for qRT-PCR analysis, and their expression is quantified in Fig. 12 (See Additional file 7 for detailed data). Compared with Normal anthers, the results showed that four genes were less expressed in HT-ms anthers at mononuclear stage and trinuclear stage (Fig. 12A-D). *TaPIP5K5* gene was 4.26 times lower and 2.05 times lower than in Normal anthers in HT-ms anthers at mononuclear stage and trinuclear stage, respectively (Fig. 12a). *TaPIP5K25* gene was 2.37 times lower and 1.51 times lower in than Normal anthers in HT-ms anthers of mononuclear stage and trinuclear stage, respectively (Fig. 12b). Similarly,compared with Normal anthers, *TaPIP5K38* gene was 4.94 times lower and 1.90 times lower in HT-ms anthers at mononuclear stage and trinuclear stage, respectively (Fig. 12c). *TaPIP5K50* gene had the same expression trend as the previous three genes, but showed an extremely significant difference at the mononuclear stage and significant difference at the trinuclear stage (Fig. 12d). The other four genes diverged in expression. Compared with Normal anthers, *TaPIP5K51* and *TaPIP5K56* were up-regulated at mononuclear stage and down-regulated at trinuclear stage, whereas *TaPIP5K52* and *TaPIP5K58* were up-regulated at both developmental stages in HT-ms anthers (Fig. 12e-h). In detail, *TaPIP5K51* and *TaPIP5K56* were 3.31 times higher and 1.71 times higher compared with Normal anthers in HT-ms anthers at the mononuclear stage, respectively, whereas, *TaPIP5K51* and *TaPIP5K56* were 1.48 times lower and 1.43 times lower compared with Normal anthers in HT-ms anthers at the trinuclear stage, respectively (Fig. 12e, g). *TaPIP5K52* and *TaPIP5K58* was 3.15 times higher and 2.95 times higher at the mononuclear stage in HT-ms anthers than in Normal anthers, respectively. At the trinuclear stage, *TaPIP5K52* showed no significant difference, however, *TaPIP5K58* was 20.65 times higher in HT-ms anthers than in Normal anthers (Fig. 12f, h).

## Discussion

With the continuous improvement of genome sequencing technology and the gradual deepening of molecular biology research, the whole genome sequencing of the heterohexaploid crop wheat has been completed, and its increasingly complete genome database has been published, which provides extremely useful resources for identifying wheat gene families, digging out functional genes and studying gene functions [29]. The phosphatidylinositol (PI) signaling system plays an important role in the process of biological development and cell response to the environment. Many studies have shown that the enzymes involved in the PI signaling system are involved in the vacuole changes during pollen development and vesicle transport during pollen tube growth [8, 30]. When the expression of these enzymes of the PI signaling system is abnormal, the vesicle transport process in the pollen tube may be affected, resulting in pollen abortion [24]. There have been studies showing that down-regulation of genes related to the PI signaling system might be the main cause of male sterility in cytoplasmic male sterility (CMS) wheat [25]. PIP5K-related genes are the key genes in the PI pathway that regulate the phosphorylation of phosphatidylinositol and the entire system of PI signaling. At present, the PIP5K gene family has been studied in various plants such as *Arabidopsis*, rice, *Glycine max*, *Phaseolus vulgaris*, *Physcomitrella patens*, and *Ginkgo biloba*, and 11, 11, 22, 12, 6 and 7 PIP5K family members have been identified, respectively [20]. However, very limited information is available regarding the PIP5K genes in wheat. In this study, a total of 64 PIP5K genes were identified in the Chinese Spring wheat genome by searching the wheat genome database, which is higher than the number of members in the above-mentioned species. The reason for this may be that wheat itself is a highly heterozygous heterohexaploid, with 3 partial homologous genomes, and there are more homoeologs. In addition, the high homology retention rate can also partially explain the large number of PIP5K genes in wheat.

The phylogenetic tree shows that 64 genes can be divided into 3 subgroups, and the motif, gene structure and domain characteristics of each subgroup obviously show conservative traits (Figs. 2 and 3). In Sub1, most PIP5K gene family members contain 7 MORN repeat motif domains and PIPKc domains, and only 8 members do not have MORN domains. The MORN domain of plants originates from an original protein. The MORN domain of plant PIP5Ks protein is significantly different from the MORN domain of other proteins [31]. In general, the N-terminal region of plant PIP5K protein has 7 or 8 MORN domains [19]. The three members in Sub2 contain typical PIP5K domains in the pfam database. Most of the family members in Sub3 contain the Cpn60_TCP1 (Chaperonin containing TCP_1 domain) domain [32] and PIPKc domain, and only 5 family members contain the Cpn60_TCP1 domain and the PIP5K domain in the pfam database. These results indicate that PIP5Ks, as a key enzyme in the PI signaling system for plant growth and development and response to environmental stress, is very conservative throughout the evolution of wheat. Moreover, the results of the Ka/Ks ratio are all less than 1 (Fig. 6), which indicates that the paralog gene pairs in the wheat PIP5K gene family have strong purifying selection, and also indicates that the wheat PIP5K gene family tends to be stable during the long-term evolution.

In this study, a collinear analysis of the PIP5K gene family among wheat chromosomes showed that the events of chromosome segment duplication occurred more frequently in the same genome group, such as 2A, 2B, and 2D. Only the 4A, 4B and 5B chromosomes had intergenomic segment replication events. To further infer the phylogenetic mechanisms of the wheat PIP5K family, we constructed two comparative syntenic maps of wheat associated with four representative species, namely rice, *Brachypodium distachyum*, millet and barley. The numbers of orthologous pairs between the other four species were 26 (rice and *Brachypodium distachyum*) and 24 (millet and barley). There were 20 orthologous pairs shared between wheat, rice, *Brachypodium distachyum* and millet (Fig. 7). These results indicate that these TaPIP5K5 genes are highly conserved. For example, *TaPIP5K1* (TraesCS2A02G130800.1), *TaPIP5K5* (TraesCS2B02G152700.1), and *TaPIP5K9* (TraesCS2D02G132600.1) showed a collinearity relationship in all five species, indicating that these genes are the most conservative. Furthermore, as a high expression gene in spikelets, roots, stems and grains in wheat, *TaPIP5K25* (TraesCS4B02G039400.2) has a collinearity relationship in wheat, rice, *Brachypodium distachyum* and millet.

Our previous results showed that male sterility could be induced by high temperature during the period of female stamens primordia formation in wheat [28]. Sterile anthers have no dehiscence, which is in sharp contrast to the obvious dehiscence of fertile anthers. Many studies have shown that there is an obvious relationship between anther dehiscence and jasmonate, for instance, MeJA/JA hormone has a strong induction effect on the spikelet opening of cereal plants such as rice, wheat and rye [33]. Other studies have shown that the levels of Ja and JA-ile increase 8.3-fold and 55-fold when rice anther dehisced compared with 2 h before dehiscence, and the expression of some genes encoding key enzymes in JA biosynthesis was also up-regulated, indicating that endogenous jasmonate played a role in anther dehiscence [34]. Recently, in thermo-sensitive genic male sterility line 4110S, researchers confirmed that high temperature treatment reduced the content of jasmonic acid in sterile anthers, and its phenotypic observation revealed that the sterile anthers had no dehiscence. It may be that the synthesis of jasmonic acid is blocked, causing the anthers to have no dehiscence and eventually leading to the occurrence of male sterility [35]. We also found that the key enzyme OPDA content in the synthesis pathway of jasmonic acid in HT-ms anthers was significantly lower than that of Normal anthers through the determination of jasmonic-related components by liquid-phase mass spectrometry. The JA-ILE level of jasmonic acid active ingredient in sterile anthers is also significantly lower than that of Normal anthers, which is consistent with the research results of Yang et al. [35] (Fig. 11). Most of the promoters of wheat PIP5K family genes involve MeJA-responsiveness and light responsiveness related elements (Fig. 8). Recent studies have also shown that components of the JA signaling pathway are involved in several light-mediated reactions [36, 37]. Therefore, it is possible that the wheat PIP5K gene family may associated with the lack of dehiscence of HT-ms anthers.

The results of qRT-PCR showed that the expression of *TaPIP5K25* in the mononuclear and trinuclear anthers of HT-ms were significantly lower than in the Normal anthers. This gene is highly conserved in the evolution of wheat, and has a collinear relationship with rice, *Brachypodium distachyum* and millet. Moreover, the expression of *TaPIP5K5*, *TaPIP5K25*, *TaPIP5K38* and *TaPIP5K50* displayed a very similar trend in the mononuclear and trinuclear stages of Normal anthers and HT-ms anthers (Fig. 12a-d). Interestingly, the domain organization of *TaPIP5K5*, *TaPIP5K25*, and *TaPIP5K50* is the structure of Subgroup 1, which is composed of MORN and PIPKc domains. TaPIP5K38 is divided into Subgroup 2 with only one PIP5K pfam domain (Fig. 3). Moreover, compared with Normal anthers in the mononuclear and trinuclear stages, the expression levels of *TaPIP5K51 and TaPIP5K56* in sterile anthers were significantly up-regulated and down-regulated in the two stages, respectively. The expression levels of *TaPIP5K52* and *TaPIP5K58* in sterile anthers were up-regulated in the two different stages (Fig. 12e-h). The similarity to the expression trend of *TaPIP5K51,56* and *TaPIP5K52,58* is consistent with the expression levels of these genes in various tissues in the RNA-seq database (Fig. 9) It is noteworthy that the four genes are divided into Subgroup 3 on domain organization, which is composed of Cpn60_TCP1 domain and PIPKc domain (Fig. 3). These results fully indicate that the differentiation of gene expression may be related to the different structural domains contained in genes. Furthermore, *TaPIP5K51*,*56*,*58* also have a collinear relationship in rice, *Brachypodium distachyum* and millet. The decrease in the expression level of some conservative genes in sterile anthers may be related to the decrease in the content of jasmin-related components. In addition, the abnormal expression of these genes may affect the entire PI signaling system, which plays a critical role in the occurrence of male sterility in wheat. In fact, results from isonuclear alloplasmic male sterile lines had already suggested that the down-regulation of gene expression related to the PI signaling system might be the main cause of male sterility in wheat anthers [25].

## Conclusions

Sixty-four TaPIP5K genes were identified on the genome of wheat. Gene structure, protein motifs, cis-acting elements, Ka/Ks analysis and the expression pattern indicated the conservative and diversified nature of TaPIP5K genes. Here, the obvious feature of HT-ms anthers is that the anthers have no dehiscence, and our determination of OPDA and JA-ILE levels proves that the OPDA content and JA-ILE content of the HT-ms anthers at the trinucleus stage are significantly lower than the Normal anthers at trinucleus stage. The qRT-PCR results showed that the expression levels of the conservative genes *TaPIP5K5*, *TaPIP5K25*, *TaPIP5K38*, *TaPIP5K50*, *TaPIP5K51*, and *TaPIP5K56* in HT-ms anthers were significantly lower than those of Normal anthers in the trinuclear stage. These results indicate that the wheat PIP5K gene family may be associated with male sterility induced by high temperature, and the upstream promoters of these genes are potentially related to jasmin. The reduction of JA-ILE levels and the low levels of these genes expression may be the main reason why HT-ms anthers have no dehiscence, ultimately leading to the abortion of the anthers.

## Methods

### Plant materials

The wheat cultivar Zhoumai 36 was seeded at the Experimental Field of Zhoukou Normal University on 13 October 2020. When Zhoumai 36 grew to the differentiation stage of pistil and stamen primordium, it was covered with a light-transmitting plastic film and subjected to high temperature stress. The procedures were performed as previous description [28]. There were 3 replicates for each sample. All samples were quick-frozen in liquid nitrogen and finally stored at − 80 °C for hormone determination and qRT-PCR. In addition, the anther materials were fixed in FAA fixative (50% ethanol, 10% formalin, and 5% acetic acid) for 1–3 days, then transferred to 70% alcohol and stored at 4 °C. The fixed anther material used conventional paraffin slice technology, and the slice thickness was set to 12 μm. After staining with Safranin-Fast Green, images were collected with an optical microscope (Nikon ECLIPSE E600). Using KI-I_2_ staining technique, the whole wheat anthers and pollen grains were stained and identified, the starch accumulation was observed, and the fertility was determined.

### Identification and characteristics of the PIP5K genes in wheat

The Hidden Markov Model (HMM) (PF01504) corresponding to the pfam PIP5K gene family was downloaded from Pfam 31.0 (http://pfam.xfam.org/) to identify the PIP5K genes in wheat. The predicted proteins in the wheat genome (IWGSC RefSeq v1.1, International Wheat Genome Sequencing Consortium website: https://wheat-urgi.versailles.inra.fr/Seq-Repository/Assemblies) were identified using HMMER v3.0. Using the original protein obtained by PIP5K HMM, a high-quality protein collection (*E*-value < 1 × 10^− 20^ and manual verification of an intact PIP5K domain) was compared, and a wheat-specific PIP5K HMM was constructed using HMMER v3.0 kit hmmbuild. This new wheat-specific PIP5K HMM was used to select all proteins with an *E*-value lower than 0.01. The obtained PIP5K gene family proteins sequence was detected on Pfam (http://pfam.xfam.org/) and CDD (https://www.ncbi.nlm.nih.gov/cdd) to ensure that these proteins contained the conserved domain unique to PIP5K. The Compute pI/MW tool on the ExPasy website (http://au.expasy.org/tool.html) was used to analyze the amino acid sequence of the obtained PIP5K gene family proteins, and various physical and chemical properties of the PIP5K protein were obtained, including amino acid length, CDS length, molecular weight, and isoelectric point.

### Phylogenetic analysis of wheat PIP5K gene family

The PIP5K protein sequences of *Arabidopsis*, soybean, rice, kidney bean, and corn were retrieved and downloaded from the TAIR (https://www.arabidopsis.org) and EnsemblPlants (http://plants.ensembl.org/index.html) databases, respectively. MEGA-X software was used to compare the identified wheat PIP5K amino acid sequence with the related sequences of *Arabidopsis*, *Glycine max*, rice, *Phaseolus vulgaris*, and maize to construct a phylogenetic tree: Neighbor-Joining algorithm, poission correction, pairwise deletion, bootstrap repeated value 1000 times. The subfamily classification results of the *Arabidopsis* PIP5K gene family were referred to in order to classify the wheat PIP5K gene family.

### Analysis of exon and intron structure, protein domain and conserved motif of PIP5K

According to the annotation information of the wheat genome, the biolinux system was used to convert the wheat gff3 file to the gtf file, and obtain the UTR region, exon and intron structure information of the required PIP5K gene, and TBtools was used for visualization [38]. Conserved PIP5K sequences were identified using MEME (Multiple Em for Motif Elicitation) suite analysis (http://meme-suite.org/tools/meme). The parameters were set as follows: Each sequence could comprise any number of non-overlapping occurrences of each motif, the number of different motifs was 20, and motif length ranged from 6 to 200 amino acids. Subsequently, these files were optimized using TBtools [38].

### Cis-element prediction

Based on the chromosome location information of the wheat genome, the Gtf/Gff3 sequence extraction tool (Gtf/Gff3 sequences Extract) in the TBtools software was used to extract the 2000 bp promoter sequence upstream of the CDS of the wheat gene family member, and the extracted sequence was submitted to PlantCare (http://bioinformatics.psb.ugent.be/webtools/plantcare/html/) for cis-element prediction. Furthermore, the biological sequence viewing tool (BioSequence viewer) in TBtools was used to visualize the cis-elements of wheat PIP5K gene family members [38].

### Chromosome location and gene duplication

The Gtf/Gff3 sequences Extract tool in the TBtools software was used to extract all the CDS of wheat genes, and the Batch Traslate CDS to Protein tool was used to translate them into proteins, and then blast alignment was performed on these proteins. The comparison results were used to screen the corresponding tandem duplications and fragment duplications of the PIP5K gene family. Based on the chromosome location information of the wheat genome, the Gene Location Visualize tool and Advanced Circos in the TBtools software were used to draw the chromosome location map and Circos map.

### Collinearity analysis among species

All protein sequences and gff3 files of wheat, rice, brochypodium distachyon, millet and barley were downloaded from the EnsemblPlants database. The Blast tool in the TBtools software was used for two-way comparison. MCscanX Wrapper was run [39] to get the location information and collinearity blocks of all genes (Minimum block size was set to 30). The Multiple Synteny Plot tool in the TBtools software was used to draw the collinearity comparison map of wheat, rice and *brochypodium distachyon* and the collinearity comparison map of wheat, millet and barley.

### Determination of endogenous hormones in wheat anthers

To extract the endogenous hormones in the anther samples, this experiment used the acetonitrile solution extraction method, purified the impurities by the QuEChERS method, and concentrated the samples by N_2_ purge. Subsequently, an Agilent 1290 high-performance liquid chromatograph was connected in series with the AB company’s Qtrap6500 mass spectrometer to determine the plant endogenous hormones OPDA and JA-ILE, and internal standard substances (D-JA) were added to the extract to correct the test results. OPDA standard products, JA-ILE standard products and deuterated jasmonic acid (D-JA) standard products were all purchased from Sigma. The standard curves of OPDA and JA-ILE are shown in Fig. S2.

### Expression analysis of wheat PIP5K gene family

The wheat RNA-Seq data came from WheatOmics. The tools on the website were used to download the transcriptome expression data of the wheat variety China Spring in different tissues and save the TPM (Transcripts Per Kilobase Million) value of the wheat PIP5K genes. TBtools software was used to draw the expression heat map of wheat PIP5K gene, which was used to analyze the expression pattern of wheat PIP5K gene.

### RNA isolation and qRT-PCR validation analysis

Total RNA was extracted using the Trizol method (TriQuick Reagent, Solarbio, China) from Normal and HT-ms anthers at different developmental phases, denatured in agarose gel (1%), and stained with SolarGelRed (Solarbio, China) to check the quality of the RNA. Specific primers were designed using primer premier 5.0 software for the qRT-PCR; the primer sequence details are provided in Table S1. Detailed information on the qRT-PCR protocol is described in a previous study [40].

## Supplementary Information

**Additional file 1.** Chromosomal localization of the PIP5K genes and gene duplication events.

**Additional file 2.** The list of 52 pairs repetitive events in wheat PIP5K genes and its value of Ka, Ks, and Ka/Ks ratio.

**Additional file 3.** Collinearity data of the PIP5K genes in wheat to other four species.

**Additional file 4.** Details on cis-elements in the promoters of the PIP5K genes.

**Additional file 5.** Expression data of TaPIP5Ks in different tissues obtained from RNA-seq in wheat.

**Additional file 6.** Detailed determination data for content of JA-ILE and OPDA.

**Additional file 7.** Details data of the qRT-PCR.

**Additional file 8: Figure S1.** Conserved motifs in PIP5K proteins.

**Additional file 9: Figure S2.** Standard curves for JA-ILE and OPDA.

**Additional file 10: Table S1.** The primer sequences of target genes for analysis of qRT-PCR.

## Data Availability

All data generated or analyzed in this study are included in this published article and its Additional files. The datasets generated and analyzed during the current study are available from the corresponding author on reasonable request. The TPM value of the transcriptome expression data of the wheat variety China Spring in different tissues was obtained from the WheatOmics website (http://202.194.139.32/expression/wheat.html).

## Abbreviations

CMS: Cytoplasmic male sterility
Cpn60_TCP1: Chaperonin containing TCP_1 domain
HT-ms: Male sterility induced by high temperature
H: high Expression region
HMM: Hidden Markov Model
JA-ILE: Isoleucine jasmonic acid
L: Low Expression region
Mbp: Megabase pair
MEME: Multiple Em for Motif Elicitation
MORN: Membrane occupation and recognition nexus
OPDA: 12-oxo-phytodienoic acid
PIPK: Phosphatidylinositol phosphate kinase
PIP5K: Phosphatidylinositol 4 phosphate 5-kinase
PI: Phosphatidylinositol
TPM: Per kilobase million
