# Supplementary figures and images for: Genome-wide identification of PIP5K in wheat and its relationship with anther male sterility induced by high temperature
Source: BMC Plant Biol. 2021 Dec 16;21:598. doi: 10.1186/s12870-021-03363-1 (PMC8675513; doi:10.1186/s12870-021-03363-1)

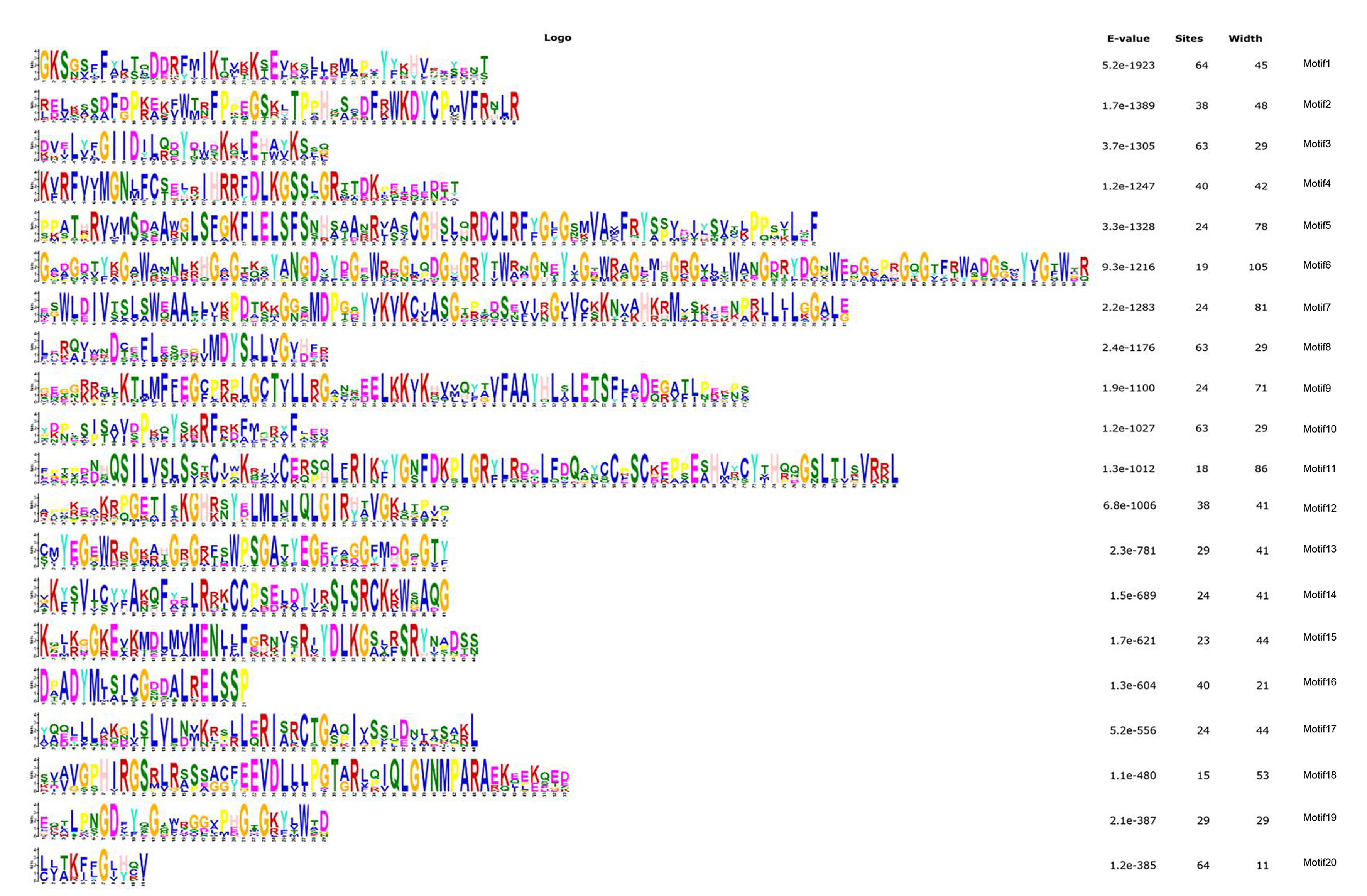

Supplement: Supplementary file 8 — Additional file 8: Figure S1. Conserved motifs in PIP5K proteins. [file 12870_2021_3363_MOESM8_ESM.tif]

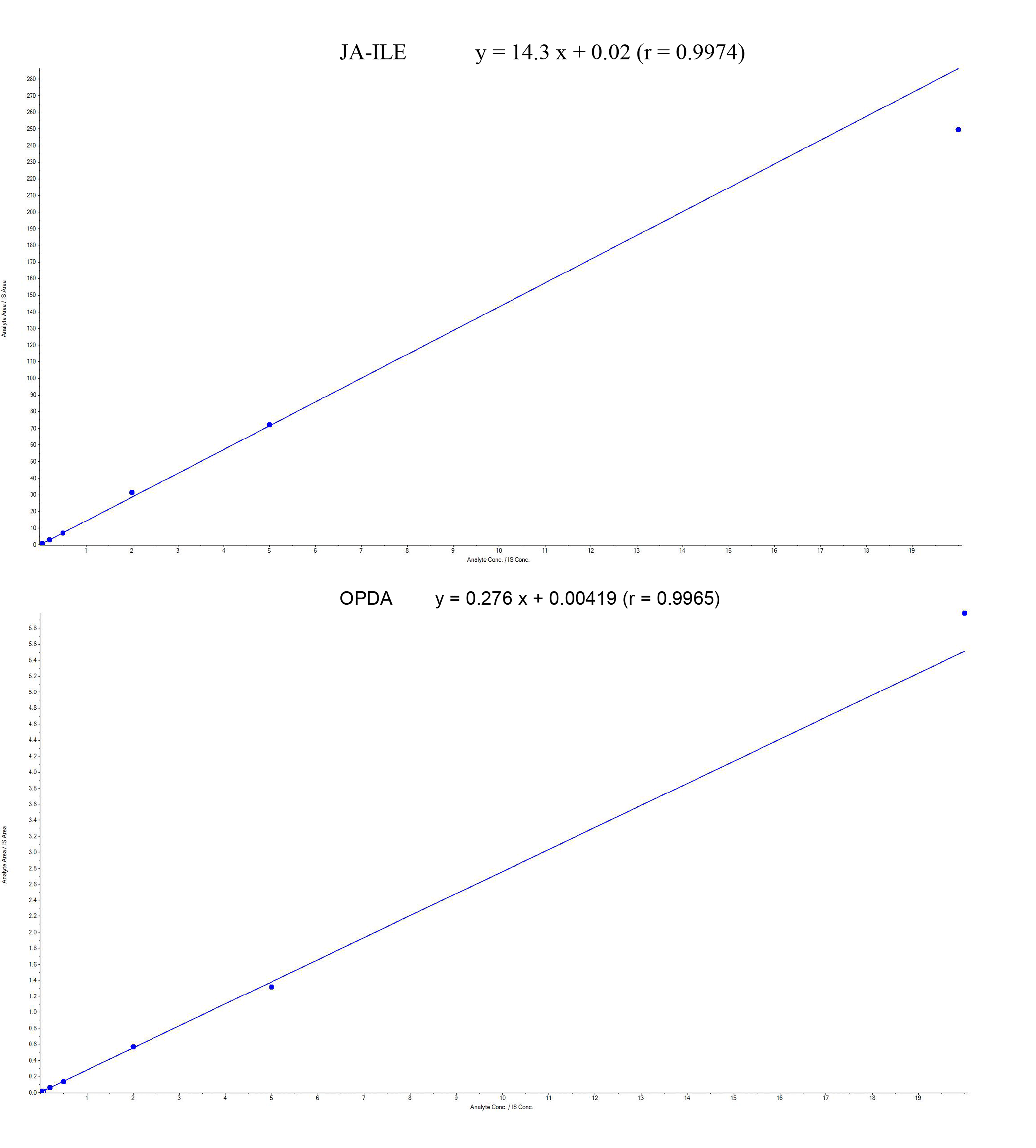

Supplement: Supplementary file 9 — Additional file 9: Figure S2. Standard curves for JA-ILE and OPDA. [file 12870_2021_3363_MOESM9_ESM.tif]
