# Supplementary material for: Genome-wide identification of PIP5K in wheat and its relationship with anther male sterility induced by high temperature
Source: BMC Plant Biol. 2021 Dec 16;21:598. doi: 10.1186/s12870-021-03363-1 (PMC8675513; doi:10.1186/s12870-021-03363-1)
Supplement: Supplementary file 10 — Additional file 10: Table S1. The primer sequences of target genes for analysis of qRT-PCR. [file 12870_2021_3363_MOESM10_ESM.docx]

Table S1 Target genes for analysis of qRT-PCR.

| Primer name | Primer sequences (5´–3´) | Primer length (bp) | Product length (bp) |
| --- | --- | --- | --- |
| TaPIP5K5-F | 5´-CAGGAAGGCTCGAAACACAC-3´ | 20 | 105 |
| TaPIP5K5-R | 5´-ATCCACCTTGAACAGCTTGC-3´ | 20 |  |
| TaPIP5K38-F | 5´-GCGGACTACATGCTTGCAAT-3´ | 20 | 132 |
| TaPIP5K38-R | 5´-CACTTCAGCCTTCTTCACGG-3´ | 20 |  |
| TaPIP5K50-F | 5´-GCTAGGGTCCAGATTCAGCT-3´ | 20 | 123 |
| TaPIP5K50-R | 5´-GTCGATGATGCCCAGGTAGA-3´ | 20 |  |
| TaPIP5K52-F | 5´-GGAGGATGCCACAGGAGAAT-3´ | 20 | 131 |
| TaPIP5K52-R | 5´-CCCGAAAATGCCCATCAACA-3´ | 20 |  |
| TaPIP5K58-F | 5´-CGCGGAATCTCTCTTTTGGG-3´ | 20 | 142 |
| TaPIP5K58-R | 5´-CGGAACATTGCAACAGTGGA-3´ | 20 |  |
| TaPIP5K51-F | 5´-GCAATACCCTGACGACGTTC-3´ | 20 | 145 |
| TaPIP5K51-R | 5´-CTCGGTGGGGTTCTCGTTAT-3´ | 20 |  |
| TaPIP5K56-F | 5´-CTCATTGAGGAGGCTGGTGA-3´ | 20 | 111 |
| TaPIP5K56-R | 5´-GCTCACATCTTCTTGGGCTG-3´ | 20 |  |
| TaPIP5K25-F | 5´-GGCTCGGTTTACAAAGGGTC-3´ | 20 | 168 |
| TaPIP5K25-R | 5´-TGACTTCCAGCTTCCGATGT-3´ | 20 |  |
| Actin-F | 5´-GGATACACGCTTCCTCATGC-3´ | 20 | 128 |
| Actin-R | 5´-CTGACAATTTCCCGCTCAGC-3´ | 20 |  |

F, forward primer; R, reverse primer; bp, base pairs.
